# DNA base pairs: the effect of the aromatic ring on the strength of the Watson–Crick hydrogen bonding†

**DOI:** 10.1039/d5ob00819k

**Published:** 2025-07-26

**Authors:** Celine Nieuwland, Michiel J. van Well, Laia Guillaumes, Anna de Vey Mestdagh, Lianne Dekker, Cynthia Nieuweboer, Sílvia Simon, Célia Fonseca Guerra

**Affiliations:** a Department of Chemistry and Pharmaceutical Sciences, Amsterdam Institute for Molecular and Life Sciences (AIMMS) Vrije Universiteit Amsterdam De Boelelaan 1108 1081 HZ Amsterdam The Netherlands c.nieuwland@vu.nl c.fonsecaguerra@vu.nl https://www.theochem.nl/; b Institut de Química Computacional i Catàlisi, Departament de Química, Universitat de Girona 17071 Girona Spain

## Abstract

The aromatic ring in DNA bases affects the Watson–Crick binding strength. Our quantum-chemical analyses, which compare the hydrogen bonding between the DNA bases and unsaturated analogs lacking the aromatic ring, reveal that this arises not from π-resonance assistance but from the electron-withdrawing (purines) or electron-donating (pyrimidines) effect of the heteroatom-containing ring on the frontier atoms. This electron redistribution modulates the electrostatics, steric Pauli repulsion, and σ-orbital interactions upon hydrogen bonding.

## Abstract 2

The aromatic ring affects the DNA Watson–Crick binding strength, not through π-resonance assistance but due to the electron-withdrawing (G and A) or electron-donating (C and T) effect on the frontier atoms.

## Graphical abstract

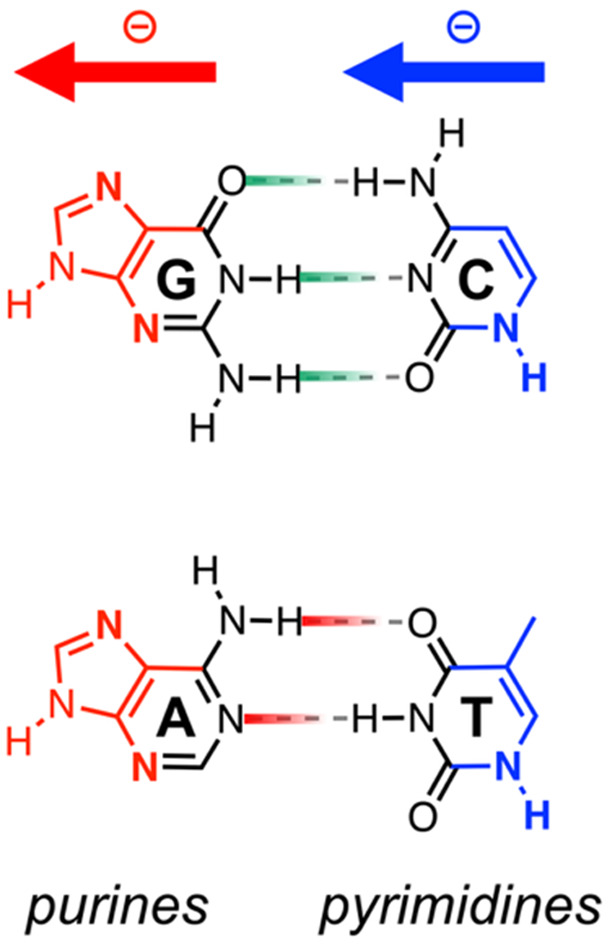

## Introduction

**Fig. 1:**
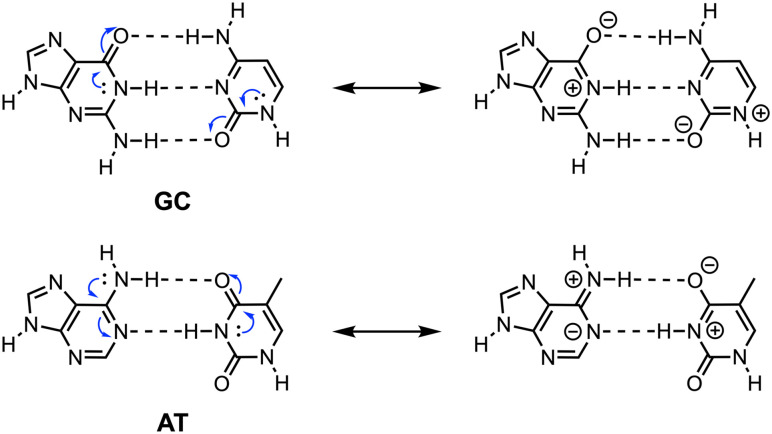
Structure of the hydrogen-bonded Watson–Crick DNA base pairs guanine–cytosine (GC) and adenine–thymine (AT) with a schematic representation of π-electron delocalization (blue arrows) leading to favorable polarization of the bases.

The DNA duplex is composed of two helical strands held together by the complementary hydrogen bonding between purine- and pyrimidine-derived nucleobases.^1^ These canonical (*i.e.*, Watson–Crick) base pairs arise from the specific hydrogen bonding between guanine (G) and cytosine (C), and adenine (A) and thymine (T) [see Fig. 1]. The hydrogen bonds result from the interaction between the frontier atoms: a partially positive *δ*^+^ N–H hydrogen-bond donor group on one nucleobase and a partially negative *δ*^−^ hydrogen-bond acceptor group (N or O) on the complementary base. Hydrogen bonds are, however, not only a pure or essentially electrostatic phenomenon but also contain a charge-transfer component of the same order of magnitude.^2^ This arises from the donor–acceptor orbital interaction between the σ-lone pair of the hydrogen-bond acceptor and the antibonding σ_NH_* orbital of the hydrogen-bond donor. The stabilizing σ-orbital interactions are in fact essential to overcome the destabilizing steric Pauli repulsion associated with hydrogen-bond formation (see ref. 2a for an overview of all relevant interaction components of hydrogen bonding).

By having three intermolecular hydrogen bonds, the consensus is that the GC pair is more stable than the AT pair, with only two hydrogen bonds. However, simply counting the number of hydrogen bonds of a base pair does not necessarily provide insight into its stability. For example, Popelier and Joubert showed that the electrostatic interactions between distant atoms also contribute to DNA base-pair stability.^3^ We have recently challenged the concept of explaining base-pair stability by the frontier atoms further, by showing that the binding strength of the GC base pair can be systematically tuned through variation of the position of the heteroatoms on the backside of the bases while keeping the frontier atoms (and their charges) unchanged.^4^ We showed that varying the position of the heteroatom-containing groups on the backside of the guanine or cytosine bases (N and NH, respectively) induces a change in the charge accumulation of the individual molecules. This affects the intermolecular binding strength through the electrostatic as well as the σ-orbital interactions. This is a manifestation of two effects: (i) electrostatic interactions are not only between the frontier atoms but also between distant atoms and (ii) molecular orbitals are delocalized over the nucleobases and their energies are therefore influenced by electronic charge changes in non-frontier parts.

The importance of considering the molecular charge accumulation rather than the charge and position of the hydrogen-bond frontier atoms was also highlighted in our previous work where we showed that the relative stabilities of multiple hydrogen-bonded dimers with the same number but different ordering of hydrogen-bond donor and acceptor groups can be explained from measuring the charge accumulation in the monomers.^5,6^

Moreover, the DNA bases are aromatic, meaning that they satisfy the Hückel criteria of being planar and having cyclic delocalization of 4*n* + 2 π electrons.^7^ So, in the DNA bases, not only the nature and position of the (hetero)atoms but also the delocalization of the π-electrons can affect the molecular charge distribution in the hydrogen-bonded monomers and thereby the stability of the base pair. This phenomenon, first proposed by Gilli *et al.* in 1989,^8*a*^ is called resonance-assisted hydrogen bonding (RAHB) and allows for favorable π-polarization of the hydrogen-bond acceptor and donor groups, which become more *δ*^−^ and *δ*^+^, respectively (schematically shown in Fig. 1), leading to an overall enforcement of the hydrogen-bond strength.^8^ The nature of the hydrogen bonds and the role of π-resonance assistance in the DNA base pairs GC and AT were studied by us in detail,^2*b*,9^ which revealed that the polarization in the π-electronic system indeed provides an additional stabilizing term to the hydrogen-bond interaction. This is, however, one order of magnitude smaller than the σ donor–acceptor orbital interactions (*vide supra*). Furthermore, it was found that there is no substantial synergistic reinforcement between the π-polarization and σ-orbital interactions and thus take place independently from each other. However, the aromatic ring and the associated π-electron delocalization are still often considered key factors in the relative binding strengths of the DNA base pairs and other aromatic multiple hydrogen-bonded systems.^10–12^

As our recent work demonstrated the relevance of non-frontier atom modifications on the molecular charge accumulation and thereby DNA base-pair stability,^4^ this inspired us to investigate the effect of the aromatic ring in the DNA bases on the strength of the Watson–Crick hydrogen bonding in more detail. To this end, the energetic components contributing to the overall binding strength of the Watson–Crick pairs GC and AT, compared to truncated analogs that lack the rear part of the aromatic ring, were investigated using dispersion-corrected density functional theory (DFT) calculations at the ZORA^13^-BLYP^14^-D3(BJ)^15^/TZ2P^16,17^ level. Our quantum-chemical analyses reveal that the binding strength of the Watson–Crick pairs is indeed influenced by the aromatic ring structure. However, we show that this effect is not due to π-resonance assistance, offering new insights into the structure and bonding of multiple hydrogen-bonding motifs for the design of innovative (bio)supramolecular building blocks.

## Results and discussion

**Fig. 2:**
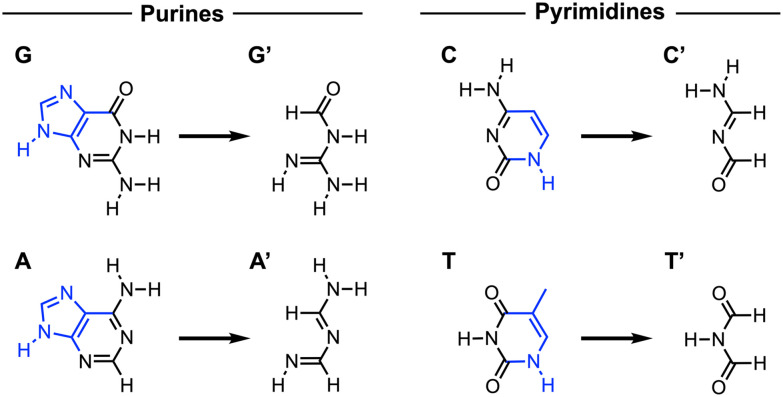
Schematic structure of the purine nucleobases guanine (G) and adenine (A) and the pyrimidine nucleobases cytosine (C) and thymine (T) and their truncated, unsaturated analogs (G′, A′, C′, and T′, respectively) studied in this work to examine the effect of the aromatic ring on the Watson–Crick hydrogen bonding. The non-aromatic analogs were obtained by removal of distant atoms (highlighted in blue) and termination of dissociated bonds by hydrogen atoms while keeping the frontier atoms unaffected.^18^

To investigate the effect of the aromatic ring on the Watson–Crick base pairing, we analyzed the complementary hydrogen bonding of the nucleobases G–C and A–T, along with their truncated, non-aromatic isosteres (G′, C′, A′, and T′) using gas phase dispersion-corrected density functional theory (DFT) calculations at the ZORA^13^-BLYP^14^-D3(BJ)^15^/TZ2P^16^ level using the Amsterdam Density Functional (ADF, version 2023.1)^17^ program as implemented in the Amsterdam Modeling Suite (AMS) [see ESI Method S1 for the full computational details†]. The truncated analogs were obtained by breaking the aromatic ring structure through removal of distant atoms and termination of dissociated bonds by hydrogen atoms while keeping the frontier atoms unchanged (see Fig. 2).^18^ Since the canonical base pairs GC and AT adopt planar minimum-energy structures, all eight complementary base-pair combinations [*i.e.*, GC, G′C, GC′, G′C′, AT, A′T, AT′, and A′T′] were optimized using *C*_s_ symmetry for a consistent comparison.

**Fig. 3:**
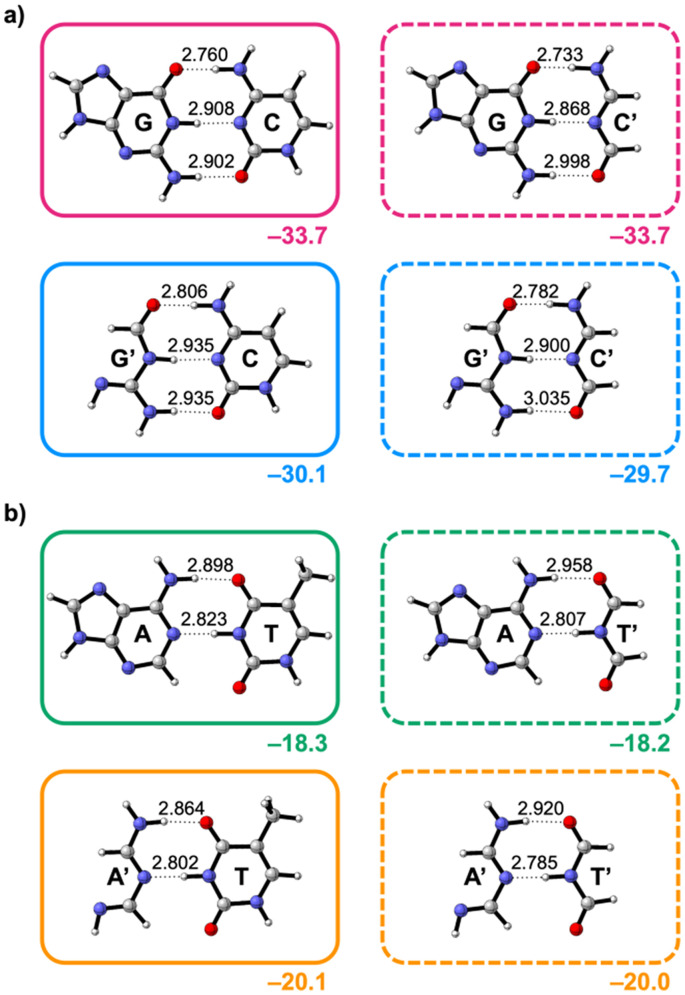
Hydrogen-bonded (a) guanine (G)–cytosine (C) and (b) adenine (A)–thymine (T) base pairs derived from the DNA bases G, C, A, and T and their non-aromatic analogs G′, C′, A′, and T′ with equilibrium hydrogen-bond [O⋯(H)N or N⋯(H)N] distances (in Å) and hydrogen-bond interaction energies Δ*E*_int_ (in kcal mol^−1^ below the demarcated areas).

The equilibrium hydrogen-bond distances and interaction energies (Δ*E*_int_) of the four GC-derived and four AT-derived base pairs are presented in Fig. 3a and b, respectively. Note that the interaction energy dictates the trend in the relative hydrogen-bond energies and is therefore regarded herein (see the hydrogen-bond energy decomposition in ESI Data S1: Table S1 and ESI Method S2 for details about this decomposition†). Our results reveal that breaking the aromatic ring in the pyrimidine bases (C and T) has little impact on Δ*E*_int_ (left to right in Fig. 3a and b). However, the removal of the aromatic ring in the purine bases G and A does affect the interaction energy Δ*E*_int_ (going from top to bottom in Fig. 3a and b). Remarkably, the effect for G and A is opposite: while Δ*E*_int_ destabilizes from G → G′, it stabilizes from A → A′. This means that the aromatic ring stabilizes the Watson–Crick hydrogen bonding of G but destabilizes that of A.

To understand this differential sensitivity towards the aromatic ring structure, Δ*E*_int_ was partitioned into four physically meaningful terms using a quantitative energy decomposition analysis (EDA).^19^ This EDA decomposes the total interaction energy Δ*E*_int_ into (i) the classical electrostatic interactions (Δ*V*_elstat_) between the unperturbed charge clouds of the two bases, (ii) the steric Pauli repulsion (Δ*E*_Pauli_) arising from the repulsion between overlapping closed-shell orbitals on the interacting bases, (iii) the orbital interaction (Δ*E*_oi_) which accounts for charge transfer (*i.e.*, donor–acceptor interactions; covalency) in the σ-electronic system (Δ*E*^σ^_oi_) and polarization of the π-electronic system (Δ*E*^π^_oi_), and (iv) the dispersion energy (Δ*E*_disp_) [see eqn (1)].

1 Δ*E*_int_ = Δ*V*_elstat_ + Δ*E*_Pauli_ + Δ*E*_oi_ + Δ*E*_disp_

Since the hydrogen-bond distances vary across the optimized base pairs (see Fig. 3), we performed the EDA at comparable hydrogen-bond distances (see ESI Data S1: Table S1 for the EDA at equilibrium distances†). This approach distinguishes inherently more stabilizing interaction terms from those enhanced by shorter distances.

**Fig. 4:**
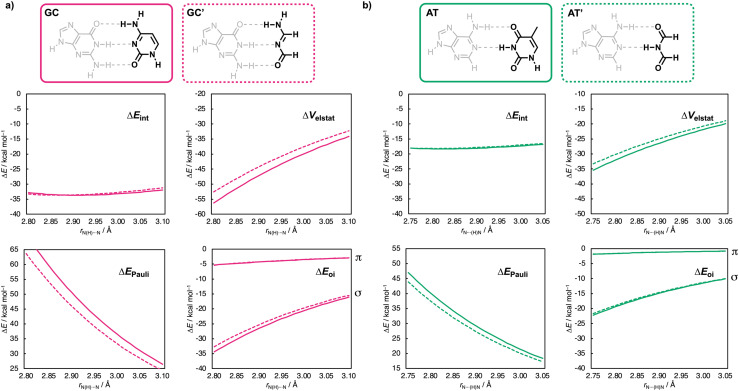
Decomposition of the hydrogen-bond interaction energy Δ*E*_int_ (in kcal mol^−1^) [Δ*E*_int_ = Δ*V*_elstat_ + Δ*E*_Pauli_ + Δ*E*_oi_ + (Δ*E*_disp_)] for (a) GC (solid magenta lines) and GC′ (dashed magenta lines) as a function of the middle hydrogen-bond distance *r*_N(H)⋯N_ (in Å, step size of 0.01 Å) and (b) for AT (solid green lines) and AT′ (dashed green lines) as a function of the lower hydrogen-bond distance *r*_N⋯(H)N_ (in Å, step size of 0.01 Å).

The EDA calculations were conducted using PyFrag 2019^20^ software, analyzing the GC-derived base pairs as a function of the middle hydrogen-bond distance, *r*_N(H)⋯N_, and the AT-derived base pairs as a function of the lower hydrogen-bond distance, *r*_N⋯(H)N_. In both cases, the two bases, in the geometry of the optimized base pair, approach each other as frozen blocks (see ESI Method S2 for details†). The EDA results, plotted in Fig. 4 and 6 (see the numerical data in ESI Data S2†), are discussed in the following sections. Notably, the dispersion energy (Δ*E*_disp_) contributes only a minor stabilizing component to the total interaction energy and remains largely unchanged for the nucleobase analogs (see ESI Data S2†). Therefore, it is not further discussed in the following sections.

### Pyrimidines

**Fig. 5:**
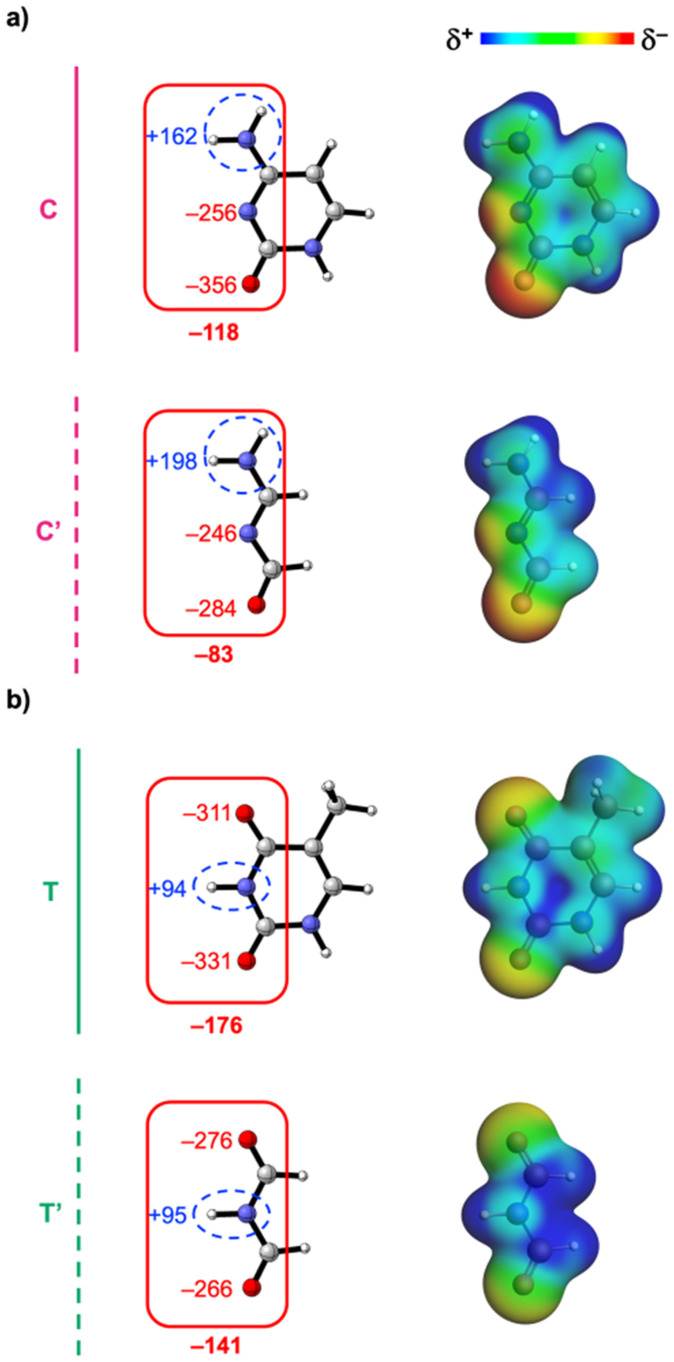
Voronoi deformation density (VDD) atomic charges *Q* (left, in milli-electrons) [sums of charges are indicated by the demarcated areas] and molecular electrostatic potential surfaces (right, at 0.01 a.u.) from −0.1 (red) to +0.1 (blue) a.u. of the isolated (a) cytosine (C and C′) and (b) thymine (T and T′) isosteres in the geometry within the base pair with the canonical complementary base, that is, G and A, respectively. Atom color code of the ball-and-stick structures: H = white; C = grey; N = blue; O = red.

We first examine why the aromatic ring has little effect on the base-pair interaction strength for the pyrimidines (Fig. 3). The EDA results as a function of the hydrogen-bond distance are presented in Fig. 4a and b for the cytosine and thymine analogs, respectively. Along the entire hydrogen-bond distance range, the interaction energy (Δ*E*_int_) remains largely unchanged when going from C → C′ or T → T′. The energy decomposition reveals that while the individual energy terms do vary, their effects counterbalance: both the destabilizing Pauli repulsion (Δ*E*_Pauli_) and stabilizing electrostatic interaction (Δ*V*_elstat_) decrease, along with a smaller reduction in stabilizing σ-orbital interactions, resulting in a nearly constant total interaction energy Δ*E*_int_. The changes in the interaction terms stem from changes in the molecular charge distribution within the individual pyrimidine bases upon removal of the aromatic ring, including a polar (non-frontier) NH group. This electron-donating NH group increases the electronic density on the front side of the pyrimidines. So, removing it upon going from C → C′ and from T → T′ causes the frontier region to become less negatively charged. This effect is evident from the Voronoi deformation density (VDD)^21^ charges and the molecular electrostatic potential surfaces shown in Fig. 5 (see ESI Method S3 for details about the VDD method and ESI Data S3: Fig. S2 for the complete VDD charge analysis†).

**Fig. 6:**
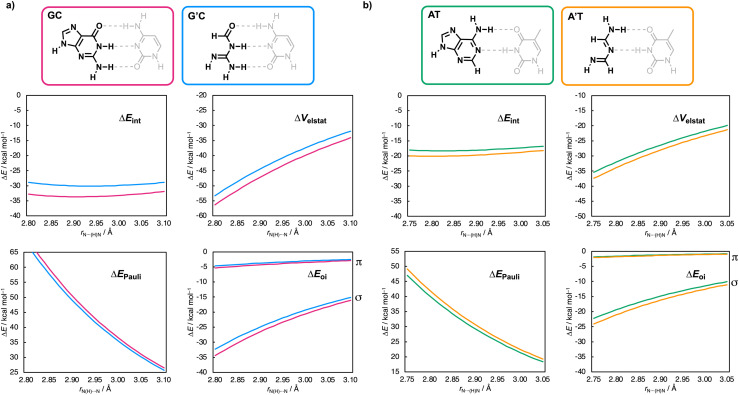
Decomposition of the hydrogen-bond interaction energy Δ*E*_int_ (in kcal mol^−1^) [Δ*E*_int_ = Δ*V*_elstat_ + Δ*E*_Pauli_ + Δ*E*_oi_ + (Δ*E*_disp_)] for (a) GC (magenta lines) and G′C (blue lines) as a function of the middle hydrogen-bond distance *r*_N(H)⋯N_ (in Å, step size of 0.01 Å) and (b) for AT (green lines) and A′T (orange lines) as a function of the lower hydrogen-bond distance *r*_N⋯(H)N_ (in Å, step size of 0.01 Å).

**Fig. 7:**
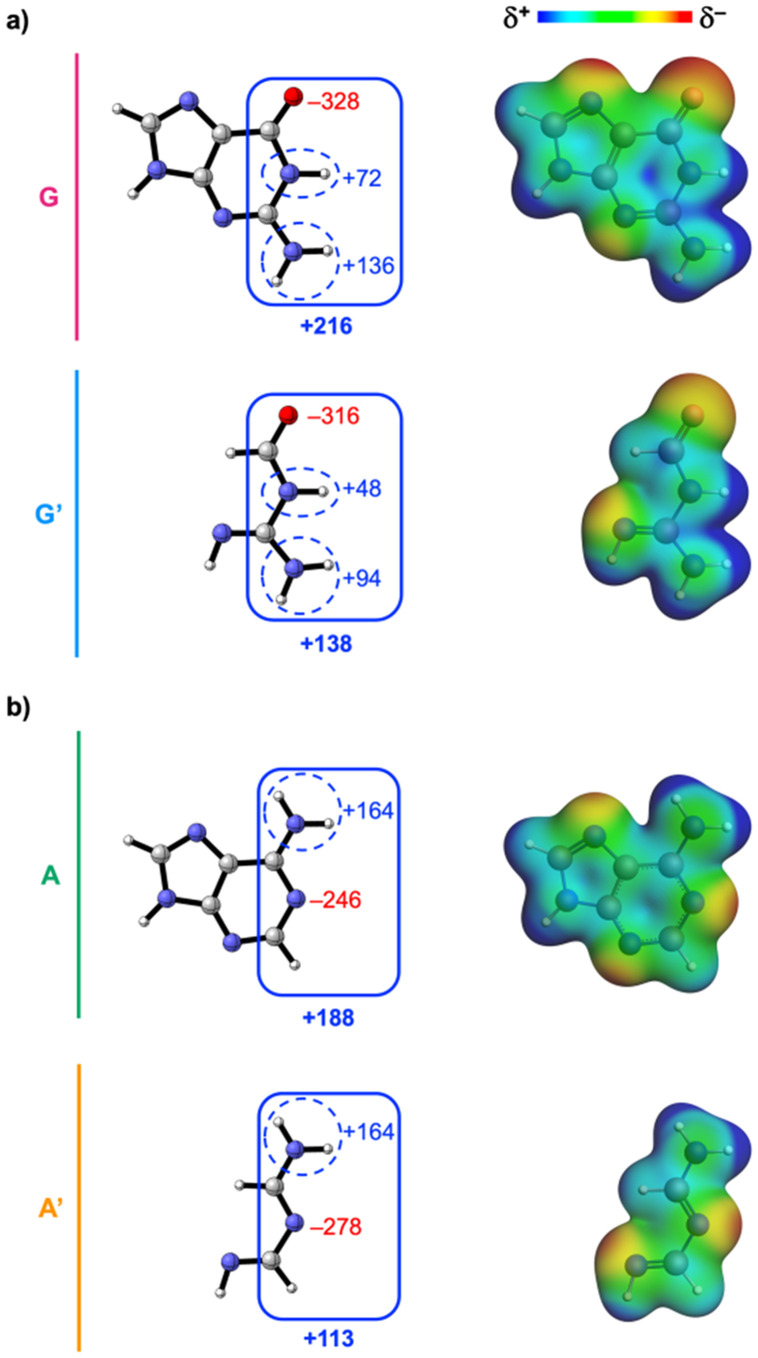
Voronoi deformation density (VDD) atomic charges *Q* (left, in milli-electrons) [sums of charges are indicated by the demarcated areas] and molecular electrostatic potential surfaces (right, at 0.01 a.u.) from −0.1 (red) to +0.1 (blue) a.u. of the isolated (a) guanine (G and G′) and (b) adenine (A and A′) isosteres in the geometry within the base pair with the canonical complementary base, that is, C and T, respectively. Atom color code of the ball-and-stick structures: H = white; C = grey; N = blue; O = red.

The reduction of the electronic density on the front side upon removal of the heteroatom-containing aromatic ring makes Δ*E*_Pauli_ less destabilizing because there is less overlap region between the filled orbitals that construct the electronic density at the hydrogen-bonding sites. Simultaneously, the less negative front side weakens the electrostatic attraction with the net positively charged front side of the purines (see Fig. 7). This effect is also reflected by the hydrogen-bond donor and acceptor groups: upon removal of the aromatic ring, the N–H donor groups become more positive, while the acceptor groups (O and N) become less negative (Fig. 5a and b). As a result, for each pyrimidine, electrostatic interactions are enhanced for the NH donor group but weakened for the acceptor groups, leading to an overall destabilization of Δ*V*_elstat_. The change in charge accumulation around the frontier atoms also influences the orbital energies (see ESI Data S3: Fig. S3†) and thereby the σ donor–acceptor interactions.^4–6^ The increased positive charge around the NH donors stabilizes the unoccupied orbitals (σ_LUMOs_) involved in the hydrogen bonding, making them better electron acceptors. On the other hand, the reduced negative charge around the O and N acceptors stabilizes their lone-pair orbitals (σ_HOMOs_), making them weaker electron donors. This only has a net destabilizing effect on the σ donor–acceptor interactions for C → C′, as the GC pair involves three σ donor–acceptor interactions (two from weakened hydrogen-bond acceptors and one from an enhanced hydrogen-bond donor). In contrast, the AT pair forms only two hydrogen bonds (from one enhanced donor and one weakened acceptor upon T → T′), so the stabilizing and destabilizing effects largely counterbalance, leading to minimal variation in Δ*E*_oi_^σ^ when going from T → T′.

Compared to the σ-orbital interactions, the π-orbital interactions (Δ*E*_oi_^π^ ) provide only a minor stabilizing contribution and, strikingly, remain unchanged in the non-aromatic pyrimidine analogs (Fig. 4). This means that the π-polarization and π-resonance assistance to the hydrogen bonding is unaffected upon reduction of the number of π-electrons and disruption of the aromatic ring of the pyrimidine bases. Instead, the aromatic ring, including the electron-donating NH group, increases the electronic density around the frontier atoms of the pyrimidine bases. This leads to more stabilizing electrostatic interactions (and to a lesser extent, enhanced covalency), while also increasing the steric Pauli repulsion upon base pairing. These opposing effects effectively cancel each other out, resulting in no net effect of the aromatic ring on the Watson–Crick pairing strength.

### Purines

Next, we examine why the aromatic ring does affect the base-pair interaction strength for the purine nucleobases (Fig. 3). The EDA results as a function of the hydrogen-bond distance are presented in Fig. 6a and b for the guanine and adenine analogs, respectively. This analysis reveals an opposing trend along the entire hydrogen-bond distance range: Δ*E*_int_ destabilizes from G → G′ but stabilizes from A → A′. This means that the aromatic ring stabilizes the Watson–Crick hydrogen bonding for guanine but destabilizes it for adenine.

The energy decomposition analysis shows that for G → G′, the destabilization in Δ*E*_int_ is mainly due to weaker electrostatic interactions (Δ*V*_elstat_) and, to a lesser extent, weaker σ-orbital interactions (Δ*E*_oi_^σ^ ). Unlike the pyrimidines, this destabilization is only partially offset by the slightly reduced Pauli repulsion (Δ*E*_Pauli_) for the truncated guanine analog (G′), leading to an overall decrease in stabilizing interaction energy Δ*E*_int_. On the other hand, for adenine (A → A′), the trend reverses: Δ*E*_int_ becomes more stabilizing due to stronger electrostatic interactions (Δ*V*_elstat_) and slightly more stabilizing σ-orbital interactions (Δ*E*^σ^_oi_). Again, this stabilization is only partially offset by the increased Pauli repulsion as one goes from A → A′.

As for the pyrimidines, the π-orbital interactions (Δ*E*_oi_^π^ ) provide only a minor stabilizing contribution and remain largely unchanged in the non-aromatic analogs. This indicates again that π-polarization and π-resonance effects on the hydrogen bonding are unaffected by the reduction of π-electrons and disruption of the aromatic ring in the purine bases.

To understand the opposing effects of the aromatic ring on hydrogen bonding of the two purine bases, we analyzed how its removal, including the heteroatoms, alters the molecular charge distribution of the individual purine molecules (see Fig. 7).

In contrast to the pyrimidines, where the electron-donating NH group increases the electronic density at the hydrogen-bonding sites, the non-frontier electron-withdrawing nitrogen atoms in the purine five- and six-membered rings cause a net reduction of the electronic density on the frontier region. As shown by the VDD charges and molecular electrostatic potential surfaces in Fig. 7, the front sides of both guanine (G → G′) and adenine (A → A′) become less positively charged, indicating an increase in electronic density upon removal of the heteroatom-containing aromatic ring.

This change in molecular charge distribution also affects the charge of the hydrogen-bonding groups (Fig. 7), thereby altering the electrostatic interactions (Δ*V*_elstat_) upon hydrogen bonding. The increased frontier electronic density upon going from G → G′ and A → A′ is reflected in the charge of the hydrogen-bond donor and acceptor groups near the nitrogen atom in the six-membered ring (Fig. 7). For guanine, the removal of the aromatic ring causes both N–H donors to become less positively charged (*i.e.*, weaker hydrogen-bond donors), while for adenine, the N acceptor becomes more negative (*i.e.*, a stronger hydrogen-bond acceptor).

For the carbonyl O hydrogen-bond acceptor group in guanine and the NH_2_ hydrogen-bond donor group in adenine, the effect is more complex. The reason for this is that, unlike pyrimidines, purines have an additional five-membered ring with another nitrogen atom that creates a more negative region around the adjacent, but not directly connected, hydrogen-bonding group (that is, the carbonyl O acceptor in G and the NH_2_ donor in A). Therefore, removing the five-membered ring causes the region around the carbonyl oxygen of guanine to become less negative (Fig. 7a), a weaker hydrogen-bond acceptor, and the NH_2_ group in adenine to become slightly more positive (Fig. 7b), and thus a better hydrogen-bond donor. As for the pyrimidines, the change in charge accumulation around the frontier atoms not only influences Δ*V*_elstat_ but also the energies of the orbitals involved in the σ donor–acceptor interactions (see ESI Data S3: Fig. S4†) and thereby Δ*E*_oi_^σ^ .

Thus, the overall effect of the aromatic ring structure on the charge of the hydrogen-bonding groups is opposite for guanine and adenine due to their different ordering of hydrogen-bond donor and acceptor groups. For G, the heteroatom-containing ring causes both NH hydrogen-bond donor groups to become more positive and the O hydrogen-bond acceptor more negative, which stabilizes the electrostatic interactions (Δ*V*_elstat_) and, to a lesser extent, the σ-orbital interactions (Δ*E*_oi_^σ^ ) upon hydrogen bonding with C. In contrast, for A, the nitrogen atom-containing ring reduces the positivity of the NH_2_ hydrogen-bond donor group and the negativity of the N hydrogen-bond acceptor, destabilizing both Δ*V*_elstat_ and Δ*E*_oi_^σ^  upon hydrogen bonding with T. So, the aromatic ring has a stabilizing effect on the Watson–Crick hydrogen bonding of guanine, while it has a destabilizing effect on that of adenine. Strikingly, this influence is not due to a change in π-resonance assistance (which remains unchanged) but caused by modulation of the electronic density at the frontier atoms driven by the non-frontier electron-withdrawing nitrogen atoms in the purine ring.

## Conclusions

The aromatic ring in DNA bases affects the Watson–Crick binding strength. This, however, does not arise from π-resonance assistance but from the electron-withdrawing (purines) or electron-donating (pyrimidines) effect of the heteroatom-containing ring on the hydrogen-bond frontier atoms, as follows from the quantum-chemical analysis of DNA base pairs with and without the aromatic ring. The electronic density redistribution caused by the heteroatom-containing ring modulates the hydrogen-bond strength through electrostatics, Pauli repulsion, and σ donor–acceptor interactions.

For pyrimidines [cytosine (C) and thymine (T)], the six-membered aromatic ring, containing an electron-donating NH group, increases the electron density at the frontier atoms. This amplifies the steric Pauli repulsion but also strengthens the electrostatic stabilization upon hydrogen bonding, resulting in no net effect of the aromatic ring on the total Watson–Crick binding strength.

On the other hand, purines [guanine (G) and adenine (A)] contain electron-withdrawing nitrogen atoms in their aromatic ring structure, which reduces the net electronic density at the frontier atoms. This redistribution mainly affects the electrostatic interactions upon hydrogen bonding, with a smaller effect on the σ donor–acceptor interactions and Pauli repulsion. Due to differences in the type and number of hydrogen-bonding groups of the two purine bases, this effect is stabilizing for guanine but destabilizing for adenine. Thus, the aromatic ring enhances the Watson–Crick hydrogen bonding for guanine but weakens it for adenine.

Strikingly, we show that removing the aromatic ring and thereby reducing the number of π-electrons does not affect the π-polarization or π-resonance assistance in the Watson–Crick hydrogen bonding. These findings provide new insights into the structure and bonding of multiple hydrogen-bonding motifs for the design of (bio)supramolecular building blocks.

## Conflicts of interest

There are no conflicts to declare.

## Supplementary Material

OB-023-D5OB00819K-s001

OB-023-D5OB00819K-s002

## Data Availability

Additional computational results that support the findings in this work, full computational details, and the Cartesian coordinates and energies of the reported molecules and hydrogen-bonded pairs have been included as part of the ESI,† which cites additional references [ref. 22–26].
