# Supplementary material for: DNA base pairs: the effect of the aromatic ring on the strength of the Watson–Crick hydrogen bonding
Source: Org Biomol Chem. 2025 Jul 26;23(43):9972–9. doi: 10.1039/d5ob00819k (PMC12314737; doi:10.1039/d5ob00819k)
Supplement: OB-023-D5OB00819K-s002 [file OB-023-D5OB00819K-s002.pdf]

This journal is © The Royal Society of Chemistry 2025

Electronic Supplementary Information (ESI)

for

DNA base pairs: The effect of the aromatic ring on the strength of the Watson-Crick hydrogen bonding

Celine Nieuwland,*[a] Michiel J. van Well,[a] Laia Guillaumes,[b] Anna de Vey Mestdagh,[a] Lianne Dekker,[a] Cynthia Nieuweboer,[a] Sílvia Simon[b] and Célia Fonseca Guerra*[a]

[a] Department of Chemistry and Pharmaceutical Sciences, Amsterdam Institute for Molecular and Life Sciences (AIMMS), Vrije Universiteit Amsterdam, De Boelelaan 1108, 1081 HZ Amsterdam, The Netherlands. E-mail: c.nieuwland@vu.nl – c.fonsecaguerra@vu.nl | Homepage: https://www.theochem.nl/

[b] Institut de Química Computacional i Catàlisi, Departament de Química, Universitat de Girona, 17071 Girona, Spain.

Table of Contents

Method S1. Computational details 2

Method S2. Analysis of the hydrogen-bond interaction 3

Method S3. Voronoi deformation density (VDD) charges 5

Data S1. Equilibrium hydrogen-bonded base pairs 6

Data S2. Complete EDA results as a function of the hydrogen-bond distance 7

Data S3. Atomic charges and orbital energies 21

Data S4. Cartesian coordinates and energies of optimized structures 24

Method S1. Computational details

All calculations were performed using the Amsterdam Density Functional (ADF, version 2023.1) program as implemented in the Amsterdam Modeling Suite (AMS).[S1] The dispersion-corrected relativistic density functional theory (DFT) calculations were performed in the gas phase at the ZORA-BLYP-D3(BJ)[S2]/TZ2P level of theory, which has been proven to be adequate for studying hydrogen-bonded base pairs.[S3] The TZ2P basis set[S4] consists of an uncontracted set of Slater-type orbitals (STOs) of triple-ζ quality for all atoms, augmented with two sets of polarization functions. The ZlmFit[S5a] density fitting scheme with the Becke[S5b] integration grid, at 'Excellent' quality, was used for all calculations. All optimized geometries have been checked to be energy minima by performing a vibrational frequency analysis (*i.e.,* no imaginary frequencies for minima, see ESI Data S4).[S6]

The energy decomposition analyses (EDA, see Method S2 for details) as a function of the hydrogen-bond distance (see Fig. 4 and 6 in the main text) were performed using the PyFrag 2019 program.[S7] The ball-and-stick structures of the optimized molecules in this work were created with the CYLview20[S8] visualization software.

[S1] (a) G. te Velde, F. M. Bickelhaupt, E. J. Baerends, C. Fonseca Guerra, S. J. A. van Gisbergen, J. G. Snijders and T. Ziegler, *J. Comput. Chem.*, 2001, **22**, 931; (b) C. Fonseca Guerra, J. G. Snijders, G. te Velde and E. J. Baerends, *Theor. Chem. Acc.*, 1998, **99**, 391; (c) ADF 2023.1, SCM, Theoretical Chemistry, Vrije Universiteit Amsterdam, Amsterdam, The Netherlands, www.scm.com; (d) AMS 2023.1, SCM, Theoretical Chemistry, Vrije Universiteit Amsterdam, Amsterdam, The Netherlands, www.scm.com.

[S2] (a) A. D. Becke, *Phys. Rev. A*, 1988, **38**, 3098; (b) C. Lee, W. Yang and R. G. Parr, *Phys. Rev. B,* 1988, **37**, 785; (c) Q. Wu and W. Yang, *J. Chem. Phys.*, 2002, **116**, 515; (d) S. Grimme, *J. Comput. Chem.,* 2004, **25**, 1463; (e) S. Grimme, *J. Comput. Chem.,* 2006, **27**, 1787; (f) S. Grimme, J. Antony, S. Ehrlich and H. Krieg, *J. Chem. Phys.,* 2010, **132**, 154104; (g) S. Grimme, S. Ehrlich and L. Goerigk, *J. Comput. Chem.,* 2011, **32**, 1456; (h) E. van Lenthe, A. Ehlers and E. J. Baerends, *J. Chem. Phys.* 1999, **110**, 8943.

[S3] (a) P. Vermeeren, L. P. Wolters, G. Paragi and C. Fonseca Guerra, *ChemPlusChem,* 2021, **86**, 812; (b) C. Fonseca Guerra, T. van der Wijst, J. Poater, M. Swart and F. M. Bickelhaupt, *Theor. Chem. Acc.,* 2010, **125**, 245; (c) C. Nieuwland, D. Almacellas, M. M. Veldhuizen, L. de Azevedo Santos, J. Poater and C. Fonseca Guerra, *Phys. Chem. Chem. Phys.,* 2024, **26**, 11306.

[S4] E. van Lenthe and E. J. Baerends, *J. Comput. Chem.,* 2003, **24**, 1142.

[S5] (a) M. Franchini, P. H. T. Philipsen, E. van Lenthe and L. Visscher, *J. Chem. Theory Comput.,* 2014, **10**, 1994; (b) A. D. Becke, *J. Chem. Phys.,* 1998, **88**, 2547; (c) M. Franchini, P. H. T. Philipsen and L. Visscher, *J. Comput. Chem.,* 2013, **34**, 1819.

[S6] (a) S. K. Wolff, *Int. J. Quantum Chem.,* 2005, **104**, 645; (b) A. Bérces, R. M. Dickson, L. Fan, H. Jacobsen, D. P. Swerhone and T. Ziegler, *Comput. Phys. Commun.,* 1997, **100**, 247; (c) H. Jacobsen, A. Bérces, D. P. Swerhone and T. Ziegler, *Comput. Phys. Commun.,* 1997, **100**, 263.

[S7] (a) X. Sun, T. M. Soini, J. Poater, T. A. Hamlin and F. M. Bickelhaupt, *J. Comput. Chem.,* 2019, **40**, 2227; (b) X. Sun, T. Soini, L. P. Wolters, W.-J. van Zeist, C. Fonseca Guerra, T. A. Hamlin and F. M. Bickelhaupt, *PyFrag 2019*, Vrije Universiteit, Amsterdam, 2019.

[S8] C. Y. Legault, *CYLview20*, Université de Sherbrooke, Sherbrooke, Quebec, Canada, **2020**, www.cylview.org.

Method S2. Analysis of the hydrogen-bond interaction

In this work, the strength of the Watson-Crick base pairing between guanine (G) – cytosine (C) and adenine (A) – thymine (T), and their truncated, non-aromatic analogs (G', C', A', and T') was examined. The hydrogen-bond energy (ΔE) of the base pairs (*i.e.,* base-pair stability) can be computed as denoted in Eq. S1.

$$\Delta E = E_{\text{basepair}} - E_{\text{base1}} - E_{\text{base2}} \qquad \text{(Eq. S1)}$$

In this equation, E_{basepair} corresponds to the electronic energy E of the hydrogen-bonded base pair, optimized in C_s symmetry, which corresponds to minimum energy structures for the canonical Watson-Crick base pairs, GC and AT (see ESI Data S4). E_{base1} and E_{base2} correspond to the electronic energies of the two separate nucleobases, each in their own unconstrained (C_1) equilibrium geometry.

To understand the different components that determine the relative stabilities of the hydrogen-bonded base pairs, ΔE was partitioned according to the activation strain model (ASM)[S9] of reactivity and bonding into a strain and interaction energy component (Eq. S2).

$$\Delta E = \Delta E_{\text{strain}} + \Delta E_{\text{int}} \qquad \text{(Eq. S2)}$$

In this decomposition, the strain energy (ΔE_{strain}) is the energy required to deform each nucleobase from its equilibrium geometry to the geometry it acquires in the hydrogen-bonded base pair. The interaction energy (ΔE_{int}) accounts for the stabilizing interaction between the two prepared (*i.e.,* deformed) bases.

ΔE_{int} can be further decomposed based on Kohn-Sham molecular orbital theory using a quantitative energy decomposition analysis (EDA).[S10] In the EDA, the total interaction energy (ΔE_{int}) is decomposed into components of electrostatic interaction (ΔV_{elstat}), Pauli repulsion (ΔE_{Pauli}), orbital interaction (ΔE_{oi}), and dispersion (ΔE_{disp}) (see Eq. S3).

$$\Delta E_{\text{int}} = \Delta V_{\text{elstat}} + \Delta E_{\text{Pauli}} + \Delta E_{\text{oi}} + \Delta E_{\text{disp}} \qquad \text{(Eq. S3)}$$

[S9] (a) P. Vermeeren, S. C. C. van der Lubbe, C. Fonseca Guerra, F. M. Bickelhaupt and T. A. Hamlin, *Nat. Protoc.,* 2020, **15**, 649; (b) F. M. Bickelhaupt and K. N. Houk, *Angew. Chem. Int. Ed.,* 2017, **56**, 10070; *Angew. Chem.,* 2017, **129**, 10204; (d) P. Vermeeren, T. A. Hamlin and F. M. Bickelhaupt, *Chem. Commun.,* 2021, **57**, 5880.

[S10] (a) F. M. Bickelhaupt and E. J. Baerends, in *Reviews in Computational Chemistry*, ed. K. B. Lipkowitz and D. B. Boyd, Wiley-VCH, New York, 2000, **15**, pp. 1–86; (b) T. A. Hamlin, P. Vermeeren, C. Fonseca Guerra, F. M. Bickelhaupt, in *Complementary Bonding Analysis*, ed. S. Grabowsky, De Gruyter, Berlin, 2021, **8**, pp. 199–212.

Here, ΔV_{elstat} comprises the (usually attractive) classical electrostatic interactions between the unperturbed charge distributions of the prepared (*i.e.,* deformed) interacting bases. ΔE_{Pauli} accounts for the destabilizing repulsive interactions arising from overlapping closed-shell orbitals and accounts for any steric repulsion. The ΔE_{oi} term comprises i) σ-charge transfer between the interacting bases (*i.e.,* donor–acceptor interactions between occupied and unoccupied orbitals on the interacting bases, including HOMO–LUMO interactions), and ii) mutual π polarization of the bases (*i.e.,* empty–occupied orbital mixing on one base due to the presence of the other base). Due to the planar (C_s) symmetry of the hydrogen-bonded base pairs, the total orbital interaction term (ΔE_{oi}) can be decomposed into these contributions stemming from the σ-charge transfer (ΔE_{oi}^{σ}) and π-polarization (ΔE_{oi}^{π}) orbital interactions (Eq. S4). Lastly, the ΔE_{disp} term includes a dispersion energy correction because of the use of Grimme's D3 dispersion correction in the computations (see Method S1 for the full computational details).

$$\Delta E_{oi} = \Delta E_{oi}^{\sigma} + \Delta E_{oi}^{\pi} \qquad \text{(Eq. S4)}$$

Besides at the equilibrium hydrogen-bond distances of the base pairs (see ESI Data S1), the interaction energies ΔE_{int} were also analyzed as a function of the middle hydrogen-bond distance $r_{N(H)\cdots N}$, for GC-derived pairs, or the lower hydrogen-bond distance $r_{N\cdots(H)N}$, for AT-derived pairs (see Fig. 4 and 6 in the main text and ESI Data S2). In this approach, the hydrogen-bond distances were varied over a certain hydrogen-bond distance interval while keeping the monomers frozen in the geometry that they acquire in the optimized base pair. The advantage of this approach is that we can compare the base pairs at similar hydrogen-bond distances while preserving the other geometrical characteristics. This allows us to differentiate between interaction terms that are intrinsically more stabilizing, from the interaction terms that are simply enhanced by the shortened hydrogen-bond distances. In other words, comparing the base pairs at similar hydrogen-bond distances allows us to identify which interaction energy term (ΔV_{elstat}, ΔE_{Pauli}, ΔE_{oi}^{σ}, ΔE_{oi}^{π}, or a combination thereof) causes the relative base-pair stabilities. This approach, in which the monomer geometries approach each other as frozen fragments, has been demonstrated before to yield identical trends compared to the approach in which the geometries of the hydrogen-bonded monomers are allowed to relax (*i.e.,* optimize) at each step of a given hydrogen-bond distance.[S11]

[S11] S. C. C. van der Lubbe, F. Zaccaria, X. Sun and C. Fonseca Guerra, *J. Am. Chem. Soc.,* 2019, **141**, 4878.

Method S3. Voronoi deformation density (VDD) charges

The Voronoi Deformation Density (VDD) charge analysis allows for the quantification of the flow of electronic charge as a direct consequence of chemical-bond formation.[S12] VDD atomic charges (Q) are computed by the spatial integration of the deformation density over the Voronoi cell of atom A, which is the space defined by the bond midplanes on and perpendicular to all bond axes between this atom A and its neighboring atoms (see Eq. S5).

$$Q = -\int_{\text{Voronoi cell of A}} [\rho(\mathbf{r}) - \textstyle\sum_i \rho_i(\mathbf{r})]\, d\mathbf{r} \qquad \text{(Eq. S5)}$$

Herein, the deformation density $\Delta\rho(\mathbf{r}) = [\rho(\mathbf{r}) - \sum_i \rho_i(\mathbf{r})]$ is the density change going from a superposition of the original atomic densities at the positions of the molecule to the actual density of that molecule. This atomic or so-called *promolecular* density is defined as the sum of the (spherically averaged) ground-state atomic densities $\sum_i \rho_i(\mathbf{r})$. This is the fictitious state in which the charge density has not been affected by chemical bonding and in which all atoms have zero charge. Q in Eq. S5 then represents the amount of charge that, due to chemical bonding, flows to a position closer to nucleus A ($Q < 0$) or to a position further away from nucleus A ($Q > 0$).

[S12] (a) C. Nieuwland, P. Vermeeren, F. M. Bickelhaupt and C. Fonseca Guerra, *J. Comput. Chem.,* 2023, **44**, 2108; (b) C. Fonseca Guerra, J. W. Handgraaf, E. J. Baerends and F. M. Bickelhaupt, *J. Comput. Chem.,* 2004, **25**, 189; (c) O. A. Stasyuk, H. Szatylowicz, T. M. Krygowski and C. Fonseca Guerra, *Phys. Chem. Chem. Phys.,* 2016, **18**, 11624.

Data S1. Equilibrium hydrogen-bonded base pairs

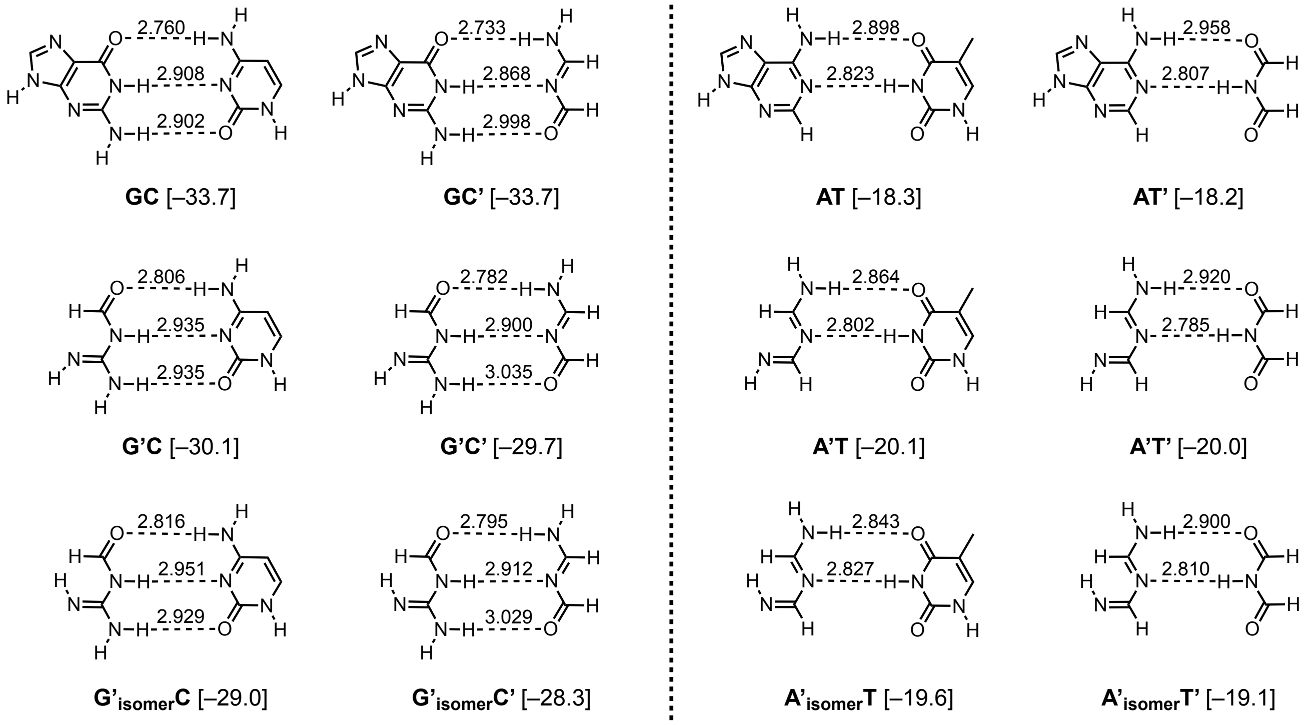

Figure S1. GC- (left) and AT-derived (right) hydrogen-bonded base pairs with equilibrium hydrogen-bond [O···(H)N or N···(H)N] distances (in Å) and interaction energies ΔE_{int} (shown in between square brackets in kcal mol^{-1}) optimized at ZORA-BLYP-D3(BJ)/TZ2P in C_s symmetry.

Table S1. Decomposition of the hydrogen-bond energy ΔE (in kcal mol^{-1}) of the planar GC- and AT-derived hydrogen-bonded base pairs that are depicted in Figure S1.[a]

	ΔE[b]	ΔE_{strain}	ΔE_{int}	ΔV_{elstat}	ΔE_{Pauli}	ΔE_{oi}[c]	ΔE_{oi}^{σ}	ΔE_{oi}^{π}	ΔE_{disp}
**GC**	–29.7	3.9	–33.7	–46.5	49.2	–30.2	–25.9	–4.2	–6.2
**G’C**	–26.6	3.5	–30.1	–41.8	43.9	–26.3	–22.8	–3.5	–5.9
**G’$_{isomer}$ C**[d]	–24.8	4.2	–29.0	–40.1	42.0	–25.0	–21.7	–3.3	–5.8
**GC’**	–29.3	4.4	–33.7	–46.7	51.0	–32.0	–27.4	–4.6	–6.0
**G’C’**	–25.9	3.8	–29.7	–41.4	44.7	–27.3	–23.6	–3.7	–5.7
**G’$_{isomer}$C’**[d]	–24.0	4.3	–28.3	–39.4	42.6	–25.9	–22.4	–3.4	–5.6
**AT**	–16.5	1.8	–18.3	–30.6	37.4	–19.8	–18.3	–1.5	–5.3
**A’T**	–17.1	3.0	–20.1	–33.8	41.8	–22.9	–21.0	–1.8	–5.2
**A’$_{isomer}$T**[d]	–15.0	4.5	–19.6	–32.4	40.0	–22.0	–20.2	–1.8	–5.2
**AT’**	–16.3	1.9	–18.2	–29.7	36.7	–20.2	–18.6	–1.6	–4.9
**A’T’**	–16.8	3.2	–20.0	–32.9	41.1	–23.3	–21.5	–1.8	–4.8
**A’$_{isomer}$T’**[d]	–14.5	4.6	–19.1	–31.1	38.9	–22.0	–20.2	–1.8	–4.8

[a] Computed at ZORA-BLYP-D3(BJ)/TZ2P. [b] Bond energies ΔE are with respect to the monomers in their unconstrained (C_1) equilibrium geometry. [c] The total orbital interaction can be decomposed into components of the σ- and π-orbital interactions due to the C_s symmetry of the base pairs: $\Delta E_{oi} = \Delta E_{oi}^{\sigma} + \Delta E_{oi}^{\pi}$. [d] The isomers of G’ and A’ (*i.e.,* G’$_{isomer}$ and A’$_{isomer}$), with the N–H bond pointing the other way, were included for completeness. However, due to the higher strain and weaker hydrogen-bond interaction in the corresponding planar base pairs, we do not focus on them in further analyses.

Data S2. Complete EDA results as a function of the hydrogen-bond distance

Table S2. The following tables contain the complete energy decomposition analysis (EDA) results (in kcal mol^{-1}) of the hydrogen-bond interaction of the GC-derived base pairs as a function of the middle hydrogen-bond distance $r_{N(H)\cdots N}$ (in Å, step size of 0.01 Å) in which the bases approach each other as frozen blocks (see Method S2 for details). All computed at ZORA-BLYP-D3(BJ)/TZ2P in C_s symmetry.

Total interaction energy ΔE_{int}				
$r_{N(H)\cdots N}$ (Å)	GC	G'C	GC'	G'C'
2.80	–32.8	–28.9	–33.3	–29.1
2.81	–33.0	–29.1	–33.4	–29.2
2.82	–33.1	–29.2	–33.5	–29.3
2.83	–33.2	–29.4	–33.6	–29.4
2.84	–33.4	–29.5	–33.6	–29.5
2.85	–33.4	–29.7	–33.6	–29.6
2.86	–33.5	–29.8	–33.6	–29.6
2.87	–33.6	–29.9	–33.7	–29.7
2.88	–33.6	–29.9	–33.6	–29.7
2.89	–33.7	–30.0	–33.6	–29.7
2.90	–33.7	–30.0	–33.6	–29.7
2.91	–33.7	–30.1	–33.5	–29.7
2.92	–33.7	–30.1	–33.5	–29.7
2.93	–33.6	–30.1	–33.4	–29.7
2.94	–33.6	–30.1	–33.4	–29.6
2.95	–33.6	–30.1	–33.3	–29.6
2.96	–33.5	–30.1	–33.2	–29.5
2.97	–33.5	–30.0	–33.1	–29.5
2.98	–33.4	–30.0	–33.0	–29.4
2.99	–33.3	–30.0	–32.9	–29.3
3.00	–33.2	–29.9	–32.7	–29.2
3.01	–33.1	–29.8	–32.6	–29.1
3.02	–33.0	–29.8	–32.5	–29.0
3.03	–32.9	–29.7	–32.4	–28.9
3.04	–32.8	–29.6	–32.2	–28.8
3.05	–32.7	–29.5	–32.1	–28.7
3.06	–32.5	–29.4	–31.9	–28.6
3.07	–32.4	–29.3	–31.7	–28.5
3.08	–32.2	–29.2	–31.6	–28.3
3.09	–32.1	–29.0	–31.4	–28.2
3.10	–31.9	–28.9	–31.2	–28.1

Electrostatic interaction ΔV_{elstat}				
$r_{N(H)\cdots N}$ (Å)	GC	G'C	GC'	G'C'
2.80	–56.3	–53.3	–52.6	–49.5
2.81	–55.3	–52.3	–51.7	–48.6
2.82	–54.3	–51.3	–50.8	–47.7
2.83	–53.3	–50.4	–49.9	–46.9
2.84	–52.4	–49.5	–49.0	–46.0
2.85	–51.5	–48.6	–48.2	–45.2
2.86	–50.6	–47.7	–47.4	–44.4
2.87	–49.7	–46.8	–46.6	–43.6
2.88	–48.8	–46.0	–45.8	–42.9
2.89	–48.0	–45.2	–45.0	–42.1
2.90	–47.2	–44.4	–44.3	–41.4
2.91	–46.4	–43.6	–43.5	–40.7
2.92	–45.6	–42.9	–42.8	–40.0
2.93	–44.8	–42.1	–42.1	–39.4
2.94	–44.1	–41.4	–41.4	–38.7
2.95	–43.3	–40.7	–40.7	–38.0
2.96	–42.6	–40.0	–40.1	–37.4
2.97	–41.9	–39.4	–39.4	–36.8
2.98	–41.2	–38.7	–38.8	–36.2
2.99	–40.6	–38.1	–38.2	–35.6
3.00	–39.9	–37.4	–37.6	–35.0
3.01	–39.3	–36.8	–37.0	–34.5
3.02	–38.7	–36.2	–36.4	–33.9
3.03	–38.0	–35.6	–35.9	–33.4
3.04	–37.4	–35.1	–35.3	–32.9
3.05	–36.9	–34.5	–34.8	–32.4
3.06	–36.3	–34.0	–34.3	–31.9
3.07	–35.7	–33.4	–33.8	–31.4
3.08	–35.2	–32.9	–33.2	–30.9
3.09	–34.7	–32.4	–32.8	–30.4
3.10	–34.1	–31.9	–32.3	–30.0

Pauli repulsion ΔE_{Pauli}				
$r_{N(H)\cdots N}$ (Å)	GC	G'C	GC'	G'C'
2.80	70.1	68.0	63.6	61.7
2.81	67.8	65.8	61.6	59.7
2.82	65.6	63.7	59.6	57.8
2.83	63.5	61.6	57.7	56.0
2.84	61.5	59.7	55.9	54.2
2.85	59.5	57.7	54.1	52.5
2.86	57.6	55.9	52.4	50.8
2.87	55.8	54.1	50.7	49.2
2.88	54.0	52.4	49.1	47.6
2.89	52.2	50.7	47.5	46.1
2.90	50.6	49.1	46.0	44.6
2.91	48.9	47.5	44.6	43.2
2.92	47.4	46.0	43.2	41.9
2.93	45.9	44.5	41.8	40.5
2.94	44.4	43.1	40.5	39.3
2.95	43.0	41.7	39.2	38.0
2.96	41.6	40.4	37.9	36.8
2.97	40.3	39.1	36.8	35.7
2.98	39.0	37.9	35.6	34.5
2.99	37.8	36.7	34.5	33.4
3.00	36.6	35.5	33.4	32.4
3.01	35.4	34.4	32.3	31.4
3.02	34.3	33.3	31.3	30.4
3.03	33.2	32.2	30.3	29.4
3.04	32.1	31.2	29.4	28.5
3.05	31.1	30.2	28.5	27.6
3.06	30.1	29.3	27.6	26.8
3.07	29.2	28.3	26.7	25.9
3.08	28.3	27.4	25.9	25.1
3.09	27.4	26.6	25.1	24.3
3.10	26.5	25.7	24.3	23.6

Total orbital interaction ΔE_{oi}				
$r_{N(H)\cdots N}$ (Å)	GC	G'C	GC'	G'C'
2.80	–39.8	–37.0	–38.0	–35.1
2.81	–38.8	–36.1	–37.0	–34.2
2.82	–37.8	–35.1	–36.1	–33.3
2.83	–36.8	–34.2	–35.2	–32.5
2.84	–35.9	–33.4	–34.3	–31.7
2.85	–35.0	–32.5	–33.4	–30.9
2.86	–34.1	–31.7	–32.6	–30.1
2.87	–33.2	–30.9	–31.8	–29.4
2.88	–32.4	–30.1	–31.0	–28.7
2.89	–31.6	–29.4	–30.3	–27.9
2.90	–30.8	–28.6	–29.5	–27.3
2.91	–30.1	–27.9	–28.8	–26.6
2.92	–29.3	–27.2	–28.1	–25.9
2.93	–28.6	–26.6	–27.4	–25.3
2.94	–27.9	–25.9	–26.8	–24.7
2.95	–27.2	–25.3	–26.1	–24.1
2.96	–26.6	–24.7	–25.5	–23.5
2.97	–25.9	–24.1	–24.9	–23.0
2.98	–25.3	–23.5	–24.3	–22.4
2.99	–24.7	–22.9	–23.7	–21.9
3.00	–24.1	–22.4	–23.2	–21.4
3.01	–23.5	–21.9	–22.6	–20.9
3.02	–23.0	–21.3	–22.1	–20.4
3.03	–22.4	–20.8	–21.6	–19.9
3.04	–21.9	–20.3	–21.1	–19.4
3.05	–21.4	–19.9	–20.6	–19.0
3.06	–20.9	–19.4	–20.1	–18.5
3.07	–20.4	–18.9	–19.7	–18.1
3.08	–19.9	–18.5	–19.2	–17.7
3.09	–19.5	–18.1	–18.8	–17.3
3.10	–19.0	–17.6	–18.3	–16.9

σ-orbital interaction ΔE_{oi}^{σ}				
$r_{N(H)\cdots N}$ (Å)	GC	G'C	GC'	G'C'
2.80	–34.4	–32.3	–32.7	–30.5
2.81	–33.5	–31.5	–31.8	–29.7
2.82	–32.7	–30.7	–31.0	–29.0
2.83	–31.8	–29.9	–30.2	–28.2
2.84	–31.0	–29.1	–29.4	–27.5
2.85	–30.2	–28.3	–28.7	–26.8
2.86	–29.4	–27.6	–28.0	–26.1
2.87	–28.6	–26.9	–27.3	–25.5
2.88	–27.9	–26.2	–26.6	–24.8
2.89	–27.2	–25.6	–25.9	–24.2
2.90	–26.5	–24.9	–25.3	–23.6
2.91	–25.8	–24.3	–24.6	–23.0
2.92	–25.2	–23.7	–24.0	–22.4
2.93	–24.6	–23.1	–23.4	–21.9
2.94	–23.9	–22.5	–22.8	–21.3
2.95	–23.3	–21.9	–22.3	–20.8
2.96	–22.8	–21.4	–21.7	–20.3
2.97	–22.2	–20.8	–21.2	–19.8
2.98	–21.6	–20.3	–20.7	–19.3
2.99	–21.1	–19.8	–20.2	–18.9
3.00	–20.6	–19.3	–19.7	–18.4
3.01	–20.1	–18.9	–19.2	–18.0
3.02	–19.6	–18.4	–18.8	–17.5
3.03	–19.1	–18.0	–18.3	–17.1
3.04	–18.6	–17.5	–17.9	–16.7
3.05	–18.2	–17.1	–17.4	–16.3
3.06	–17.8	–16.7	–17.0	–15.9
3.07	–17.3	–16.3	–16.6	–15.5
3.08	–16.9	–15.9	–16.2	–15.2
3.09	–16.5	–15.5	–15.8	–14.8
3.10	–16.1	–15.1	–15.5	–14.5

π-orbital interaction ΔE_{oi}^{π}				
$r_{N(H)\cdots N}$ (Å)	GC	G'C	GC'	G'C'
2.80	–5.4	–4.7	–5.3	–4.6
2.81	–5.2	–4.6	–5.2	–4.5
2.82	–5.1	–4.5	–5.1	–4.4
2.83	–5.0	–4.4	–5.0	–4.3
2.84	–4.9	–4.3	–4.8	–4.2
2.85	–4.8	–4.2	–4.7	–4.1
2.86	–4.7	–4.1	–4.6	–4.0
2.87	–4.6	–4.0	–4.5	–3.9
2.88	–4.5	–3.9	–4.4	–3.8
2.89	–4.4	–3.8	–4.4	–3.7
2.90	–4.3	–3.8	–4.3	–3.7
2.91	–4.2	–3.7	–4.2	–3.6
2.92	–4.1	–3.6	–4.1	–3.5
2.93	–4.1	–3.5	–4.0	–3.4
2.94	–4.0	–3.4	–3.9	–3.4
2.95	–3.9	–3.4	–3.8	–3.3
2.96	–3.8	–3.3	–3.8	–3.2
2.97	–3.7	–3.2	–3.7	–3.2
2.98	–3.7	–3.2	–3.6	–3.1
2.99	–3.6	–3.1	–3.6	–3.0
3.00	–3.5	–3.0	–3.5	–3.0
3.01	–3.5	–3.0	–3.4	–2.9
3.02	–3.4	–2.9	–3.3	–2.9
3.03	–3.3	–2.9	–3.3	–2.8
3.04	–3.3	–2.8	–3.2	–2.8
3.05	–3.2	–2.8	–3.2	–2.7
3.06	–3.1	–2.7	–3.1	–2.6
3.07	–3.1	–2.7	–3.0	–2.6
3.08	–3.0	–2.6	–3.0	–2.5
3.09	–3.0	–2.6	–2.9	–2.5
3.10	–2.9	–2.5	–2.9	–2.5

Dispersion energy ΔE_{disp}				
$r_{N(H)\cdots N}$ (Å)	GC	G'C	GC'	G'C'
2.80	–6.8	–6.6	–6.3	–6.1
2.81	–6.7	–6.5	–6.3	–6.1
2.82	–6.7	–6.5	–6.2	–6.1
2.83	–6.6	–6.4	–6.2	–6.0
2.84	–6.6	–6.4	–6.1	–6.0
2.85	–6.5	–6.3	–6.1	–5.9
2.86	–6.5	–6.3	–6.0	–5.9
2.87	–6.4	–6.2	–6.0	–5.8
2.88	–6.4	–6.2	–5.9	–5.8
2.89	–6.3	–6.1	–5.9	–5.7
2.90	–6.3	–6.1	–5.8	–5.7
2.91	–6.2	–6.0	–5.8	–5.6
2.92	–6.1	–6.0	–5.7	–5.6
2.93	–6.1	–5.9	–5.7	–5.5
2.94	–6.0	–5.9	–5.6	–5.5
2.95	–6.0	–5.8	–5.6	–5.4
2.96	–5.9	–5.8	–5.6	–5.4
2.97	–5.9	–5.7	–5.5	–5.3
2.98	–5.8	–5.7	–5.5	–5.3
2.99	–5.8	–5.6	–5.4	–5.3
3.00	–5.7	–5.6	–5.4	–5.2
3.01	–5.7	–5.5	–5.3	–5.2
3.02	–5.6	–5.5	–5.3	–5.1
3.03	–5.6	–5.4	–5.2	–5.1
3.04	–5.5	–5.4	–5.2	–5.0
3.05	–5.5	–5.3	–5.1	–5.0
3.06	–5.5	–5.3	–5.1	–4.9
3.07	–5.4	–5.2	–5.0	–4.9
3.08	–5.4	–5.2	–5.0	–4.9
3.09	–5.3	–5.1	–5.0	–4.8
3.10	–5.3	–5.1	–4.9	–4.8

Table S3. The following tables contain the complete energy decomposition analysis (EDA) results (in kcal mol^{-1}) of the hydrogen-bond interaction of the AT-derived base pairs as a function of the lower hydrogen-bond distance $r_{N\cdots(H)N}$ (in Å, step size of 0.01 Å) in which the bases approach each other as frozen blocks (see Method S2 for details). All computed at ZORA-BLYP-D3(BJ)/TZ2P in C_s symmetry.

**Total interaction energy ΔE_{int}**				
**$r_{N\cdots(H)N}$ (Å)**	**AT**	**A'T**	**AT'**	**A'T'**
2.75	–18.0	–20.0	–18.0	–19.9
2.76	–18.1	–20.0	–18.1	–19.9
2.77	–18.2	–20.1	–18.1	–20.0
2.78	–18.2	–20.1	–18.1	–20.0
2.79	–18.2	–20.1	–18.1	–20.0
2.80	–18.3	–20.1	–18.1	–20.0
2.81	–18.3	–20.1	–18.2	–19.9
2.82	–18.3	–20.1	–18.1	–19.9
2.83	–18.3	–20.1	–18.1	–19.9
2.84	–18.3	–20.0	–18.1	–19.8
2.85	–18.3	–20.0	–18.1	–19.8
2.86	–18.2	–20.0	–18.0	–19.7
2.87	–18.2	–19.9	–18.0	–19.7
2.88	–18.2	–19.9	–18.0	–19.6
2.89	–18.1	–19.8	–17.9	–19.5
2.90	–18.1	–19.7	–17.8	–19.5
2.91	–18.0	–19.6	–17.8	–19.4
2.92	–18.0	–19.6	–17.7	–19.3
2.93	–17.9	–19.5	–17.6	–19.2
2.94	–17.8	–19.4	–17.6	–19.1
2.95	–17.7	–19.3	–17.5	–19.0
2.96	–17.7	–19.2	–17.4	–18.9
2.97	–17.6	–19.1	–17.3	–18.8
2.98	–17.5	–19.0	–17.2	–18.7
2.99	–17.4	–18.9	–17.1	–18.6
3.00	–17.3	–18.8	–17.0	–18.5
3.01	–17.2	–18.7	–16.9	–18.4
3.02	–17.1	–18.5	–16.8	–18.3
3.03	–17.0	–18.4	–16.7	–18.1
3.04	–16.9	–18.3	–16.6	–18.0
3.05	–16.8	–18.2	–16.5	–17.9

Electrostatic interaction ΔV_{elstat}				
$r_{N\cdots(H)N}$ (Å)	AT	A’T	AT’	A’T’
2.75	–35.4	–37.4	–33.3	–35.2
2.76	–34.7	–36.7	–32.6	–34.6
2.77	–34.0	–36.0	–32.0	–33.9
2.78	–33.3	–35.3	–31.4	–33.2
2.79	–32.7	–34.6	–30.7	–32.6
2.80	–32.0	–33.9	–30.2	–32.0
2.81	–31.4	–33.3	–29.6	–31.4
2.82	–30.8	–32.6	–29.0	–30.8
2.83	–30.2	–32.0	–28.5	–30.2
2.84	–29.6	–31.4	–27.9	–29.7
2.85	–29.0	–30.8	–27.4	–29.1
2.86	–28.5	–30.2	–26.9	–28.6
2.87	–27.9	–29.7	–26.4	–28.0
2.88	–27.4	–29.1	–25.9	–27.5
2.89	–26.9	–28.6	–25.4	–27.0
2.90	–26.4	–28.0	–24.9	–26.5
2.91	–25.9	–27.5	–24.5	–26.0
2.92	–25.4	–27.0	–24.0	–25.6
2.93	–24.9	–26.5	–23.6	–25.1
2.94	–24.4	–26.0	–23.1	–24.6
2.95	–24.0	–25.5	–22.7	–24.2
2.96	–23.5	–25.1	–22.3	–23.8
2.97	–23.1	–24.6	–21.9	–23.3
2.98	–22.7	–24.2	–21.5	–22.9
2.99	–22.2	–23.7	–21.1	–22.5
3.00	–21.8	–23.3	–20.7	–22.1
3.01	–21.4	–22.9	–20.3	–21.7
3.02	–21.0	–22.5	–20.0	–21.4
3.03	–20.7	–22.1	–19.6	–21.0
3.04	–20.3	–21.7	–19.3	–20.6
3.05	–19.9	–21.3	–18.9	–20.3

Pauli repulsion ΔE_{Pauli}				
$r_{N\cdots(H)N}$ (Å)	AT	A’T	AT’	A’T’
2.75	47.0	49.1	43.9	45.8
2.76	45.6	47.6	42.5	44.4
2.77	44.1	46.1	41.2	43.1
2.78	42.8	44.7	39.9	41.7
2.79	41.4	43.3	38.7	40.5
2.80	40.1	42.0	37.5	39.2
2.81	38.9	40.7	36.3	38.0
2.82	37.7	39.4	35.2	36.8
2.83	36.5	38.2	34.1	35.7
2.84	35.4	37.0	33.1	34.6
2.85	34.3	35.9	32.0	33.6
2.86	33.2	34.8	31.1	32.5
2.87	32.2	33.7	30.1	31.5
2.88	31.2	32.7	29.2	30.6
2.89	30.2	31.7	28.3	29.6
2.90	29.3	30.7	27.4	28.7
2.91	28.4	29.8	26.6	27.9
2.92	27.5	28.9	25.8	27.0
2.93	26.7	28.0	25.0	26.2
2.94	25.9	27.1	24.2	25.4
2.95	25.1	26.3	23.5	24.6
2.96	24.3	25.5	22.7	23.9
2.97	23.5	24.7	22.1	23.1
2.98	22.8	23.9	21.4	22.4
2.99	22.1	23.2	20.7	21.8
3.00	21.4	22.5	20.1	21.1
3.01	20.8	21.8	19.5	20.5
3.02	20.1	21.2	18.9	19.8
3.03	19.5	20.5	18.3	19.2
3.04	18.9	19.9	17.8	18.7
3.05	18.4	19.3	17.2	18.1

Total orbital interaction ΔE_{oi}				
$r_{N\cdots(H)N}$ (Å)	**AT**	**A'T**	**AT'**	**A'T'**
2.75	–24.1	–26.3	–23.5	–25.6
2.76	–23.4	–25.6	–22.9	–24.9
2.77	–22.8	–24.9	–22.3	–24.3
2.78	–22.2	–24.2	–21.7	–23.6
2.79	–21.6	–23.6	–21.1	–23.0
2.80	–21.0	–23.0	–20.6	–22.4
2.81	–20.5	–22.4	–20.0	–21.8
2.82	–19.9	–21.8	–19.5	–21.3
2.83	–19.4	–21.2	–19.0	–20.7
2.84	–18.9	–20.7	–18.5	–20.2
2.85	–18.4	–20.1	–18.0	–19.7
2.86	–17.9	–19.6	–17.6	–19.2
2.87	–17.4	–19.1	–17.1	–18.7
2.88	–17.0	–18.6	–16.7	–18.2
2.89	–16.5	–18.1	–16.2	–17.8
2.90	–16.1	–17.6	–15.8	–17.3
2.91	–15.7	–17.2	–15.4	–16.9
2.92	–15.3	–16.8	–15.0	–16.5
2.93	–14.9	–16.3	–14.7	–16.1
2.94	–14.5	–15.9	–14.3	–15.7
2.95	–14.1	–15.5	–13.9	–15.3
2.96	–13.8	–15.1	–13.6	–14.9
2.97	–13.4	–14.7	–13.2	–14.5
2.98	–13.1	–14.4	–12.9	–14.2
2.99	–12.7	–14.0	–12.6	–13.8
3.00	–12.4	–13.6	–12.3	–13.5
3.01	–12.1	–13.3	–12.0	–13.2
3.02	–11.8	–13.0	–11.7	–12.8
3.03	–11.5	–12.6	–11.4	–12.5
3.04	–11.2	–12.3	–11.1	–12.2
3.05	–10.9	–12.0	–10.8	–11.9

σ-orbital interaction ΔE_{oi}^{σ}				
$r_{N\cdots(H)N}$ (Å)	**AT**	**A'T**	**AT'**	**A'T'**
2.75	–22.2	–24.1	–21.7	–23.5
2.76	–21.6	–23.5	–21.1	–22.9
2.77	–21.1	–22.9	–20.5	–22.3
2.78	–20.5	–22.3	–20.0	–21.7
2.79	–20.0	–21.7	–19.5	–21.2
2.80	–19.4	–21.1	–19.0	–20.6
2.81	–18.9	–20.6	–18.5	–20.1
2.82	–18.4	–20.0	–18.0	–19.6
2.83	–17.9	–19.5	–17.5	–19.1
2.84	–17.5	–19.0	–17.1	–18.6
2.85	–17.0	–18.5	–16.6	–18.1
2.86	–16.5	–18.0	–16.2	–17.7
2.87	–16.1	–17.6	–15.8	–17.2
2.88	–15.7	–17.1	–15.4	–16.8
2.89	–15.3	–16.7	–15.0	–16.4
2.90	–14.9	–16.2	–14.6	–16.0
2.91	–14.5	–15.8	–14.3	–15.6
2.92	–14.1	–15.4	–13.9	–15.2
2.93	–13.8	–15.0	–13.5	–14.8
2.94	–13.4	–14.6	–13.2	–14.4
2.95	–13.1	–14.3	–12.9	–14.1
2.96	–12.7	–13.9	–12.6	–13.7
2.97	–12.4	–13.6	–12.2	–13.4
2.98	–12.1	–13.2	–11.9	–13.1
2.99	–11.8	–12.9	–11.6	–12.7
3.00	–11.5	–12.6	–11.3	–12.4
3.01	–11.2	–12.2	–11.1	–12.1
3.02	–10.9	–11.9	–10.8	–11.8
3.03	–10.6	–11.6	–10.5	–11.5
3.04	–10.4	–11.4	–10.3	–11.3
3.05	–10.1	–11.1	–10.0	–11.0

π-orbital interaction ΔE_{oi}^{π}				
**$r_{N\cdots(H)N}$ (Å)**	**AT**	**A'T**	**AT'**	**A'T'**
2.75	–1.9	–2.1	–1.8	–2.0
2.76	–1.8	–2.1	–1.8	–2.0
2.77	–1.8	–2.0	–1.7	–1.9
2.78	–1.7	–2.0	–1.7	–1.9
2.79	–1.7	–1.9	–1.6	–1.8
2.80	–1.6	–1.8	–1.6	–1.8
2.81	–1.6	–1.8	–1.5	–1.7
2.82	–1.5	–1.7	–1.5	–1.7
2.83	–1.5	–1.7	–1.5	–1.6
2.84	–1.4	–1.7	–1.4	–1.6
2.85	–1.4	–1.6	–1.4	–1.6
2.86	–1.4	–1.6	–1.3	–1.5
2.87	–1.3	–1.5	–1.3	–1.5
2.88	–1.3	–1.5	–1.3	–1.4
2.89	–1.3	–1.4	–1.2	–1.4
2.90	–1.2	–1.4	–1.2	–1.4
2.91	–1.2	–1.4	–1.2	–1.3
2.92	–1.2	–1.3	–1.2	–1.3
2.93	–1.1	–1.3	–1.1	–1.3
2.94	–1.1	–1.3	–1.1	–1.2
2.95	–1.1	–1.2	–1.1	–1.2
2.96	–1.0	–1.2	–1.0	–1.2
2.97	–1.0	–1.2	–1.0	–1.1
2.98	–1.0	–1.1	–1.0	–1.1
2.99	–1.0	–1.1	–1.0	–1.1
3.00	–0.9	–1.1	–0.9	–1.1
3.01	–0.9	–1.1	–0.9	–1.0
3.02	–0.9	–1.0	–0.9	–1.0
3.03	–0.9	–1.0	–0.9	–1.0
3.04	–0.8	–1.0	–0.9	–1.0
3.05	–0.8	–1.0	–0.8	–0.9

Dispersion energy ΔE_{disp}				
$r_{N\cdots(H)N}$ (Å)	**AT**	**A’T**	**AT’**	**A’T’**
2.75	–5.6	–5.4	–5.1	–5.0
2.76	–5.6	–5.4	–5.1	–4.9
2.77	–5.5	–5.3	–5.1	–4.9
2.78	–5.5	–5.3	–5.0	–4.8
2.79	–5.4	–5.2	–5.0	–4.8
2.80	–5.4	–5.2	–4.9	–4.8
2.81	–5.3	–5.2	–4.9	–4.7
2.82	–5.3	–5.1	–4.8	–4.7
2.83	–5.2	–5.1	–4.8	–4.6
2.84	–5.2	–5.0	–4.8	–4.6
2.85	–5.2	–5.0	–4.7	–4.6
2.86	–5.1	–4.9	–4.7	–4.5
2.87	–5.1	–4.9	–4.6	–4.5
2.88	–5.0	–4.9	–4.6	–4.4
2.89	–5.0	–4.8	–4.6	–4.4
2.90	–4.9	–4.8	–4.5	–4.4
2.91	–4.9	–4.7	–4.5	–4.3
2.92	–4.8	–4.7	–4.4	–4.3
2.93	–4.8	–4.6	–4.4	–4.2
2.94	–4.8	–4.6	–4.4	–4.2
2.95	–4.7	–4.6	–4.3	–4.2
2.96	–4.7	–4.5	–4.3	–4.1
2.97	–4.6	–4.5	–4.2	–4.1
2.98	–4.6	–4.4	–4.2	–4.1
2.99	–4.5	–4.4	–4.2	–4.0
3.00	–4.5	–4.4	–4.1	–4.0
3.01	–4.5	–4.3	–4.1	–3.9
3.02	–4.4	–4.3	–4.0	–3.9
3.03	–4.4	–4.2	–4.0	–3.9
3.04	–4.3	–4.2	–4.0	–3.8
3.05	–4.3	–4.2	–3.9	–3.8

Data S3. Atomic charges and orbital energies

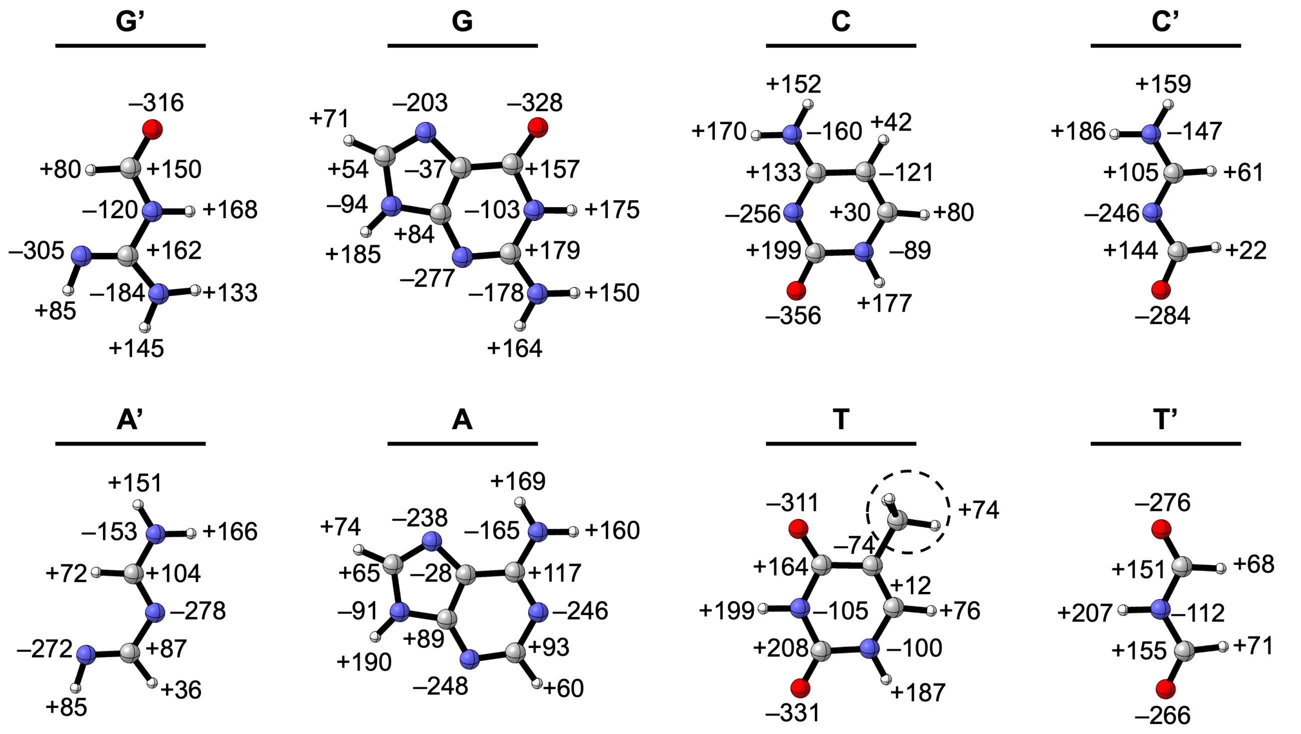

Figure S2. Voronoi deformation density (VDD) atomic charges *Q* (in milli-electrons) of the isolated guanine (G and G’), adenine (A and A’), cytosine (C and C’), and thymine (T and T’) derived bases in the geometry within the base pair with the canonical complementary nucleobase (*i.e.,* C, T, G, and A, respectively). Note that the atomic charges of the methyl group in T are summed. Computed at ZORA-BLYP-D3(BJ)/TZ2P. Atom color code of the ball-and-stick structures: H = white; C = grey; N = blue; O = red.

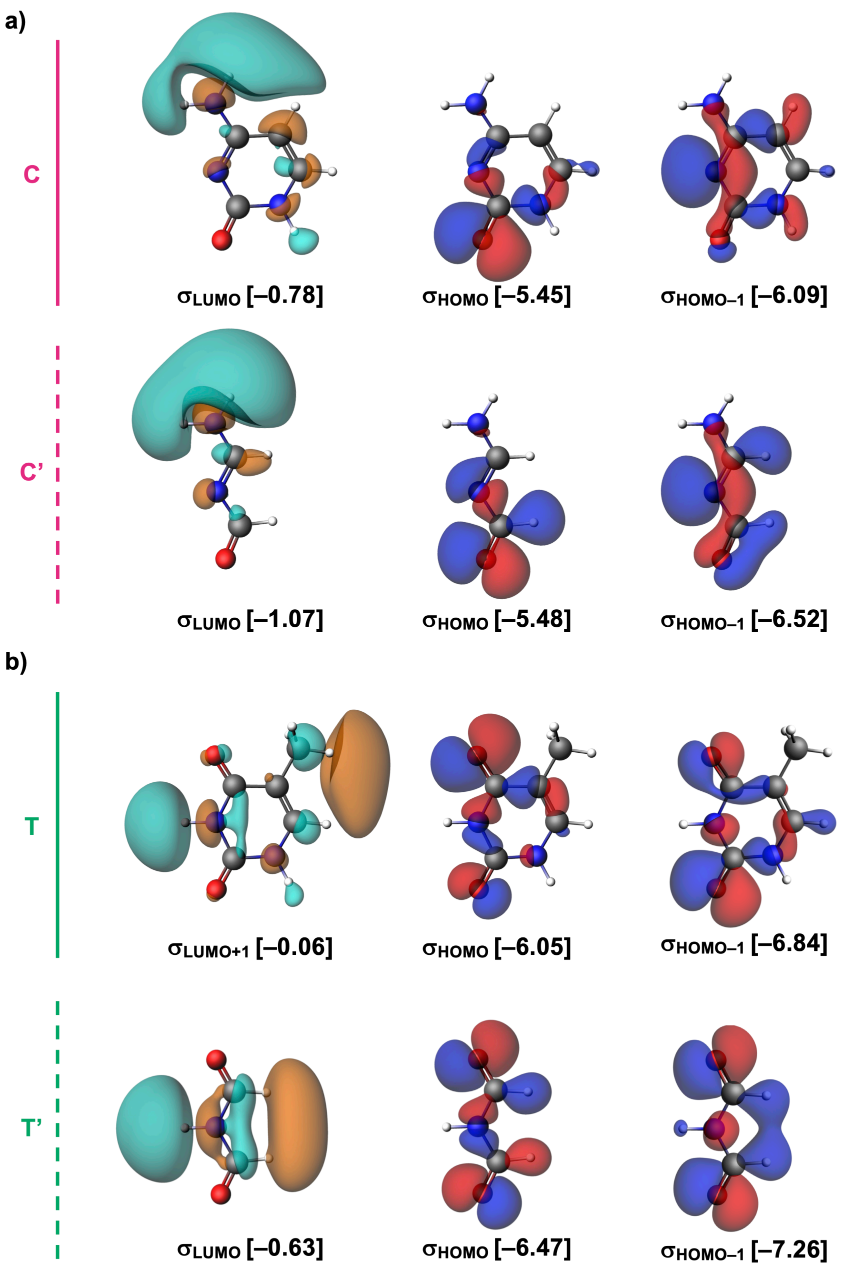

Figure S3. Isosurfaces (at 0.03 a.u.) with corresponding energies ε (in between square brackets in eV) of the unoccupied (σ_{LUMO}) and occupied (σ_{HOMO}) orbitals relevant for the Watson-Crick hydrogen bonding of the isolated (a) cytosine (C and C’) and (b) thymine (T and T’) isosteres in the geometry within the base pair with the canonical complementary base, that is, G and A, respectively. Atom color code of the ball-and-stick structures: H = white; C = grey; N = blue; O = red.

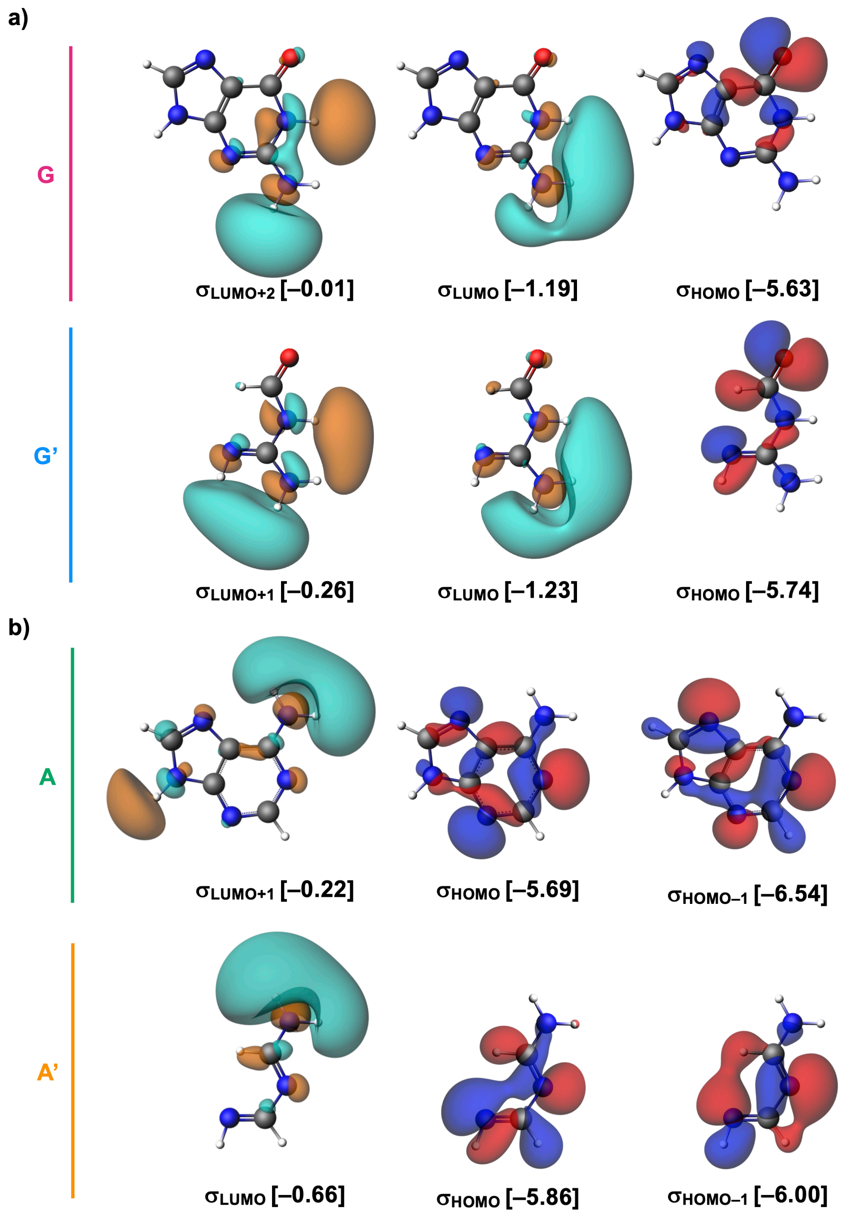

Figure S4. Isosurfaces (at 0.03 a.u.) with corresponding energies ε (in between square brackets in eV) of the unoccupied (σ_{LUMO}) and occupied (σ_{HOMO}) orbitals relevant for the Watson-Crick hydrogen bonding of the isolated (a) guanine (G and G’) and (b) adenine (A and A’) isosteres in the geometry within the base pair with the canonical complementary base, that is, C and T, respectively. Atom color code of the ball-and-stick structures: H = white; C = grey; N = blue; O = red.

Data S4. Cartesian coordinates and energies of optimized structures

Below is a list consisting of the Cartesian coordinates (in Å), ADF total bond energies *E*, enthalpies *H*, Gibbs free energies *G* (in kcal mol^{-1}), and the number of imaginary frequencies (N_{imag}) of all reported structures, optimized at the ZORA-BLYP-D3(BJ)/TZ2P level. Monomers were optimized without geometry constraints (C_1 symmetry), while the hydrogen-bonded dimers were optimized in C_s symmetry.

GC

E: −4396.42
H: −4254.65
G: −4291.92
N_{imag} = 0

```
N      0.59867360     -2.59561477      0.00000000
O      1.05345130      2.01711809      0.00000000
N      0.92872479     -0.28881274      0.00000000
C      1.47019227     -1.55836775      0.00000000
N      2.78382835     -1.78973229      0.00000000
C      3.51388229     -0.65337141      0.00000000
C      3.07174579      0.68421039      0.00000000
C      1.66043405      0.92575466      0.00000000
N      4.89206364     -0.56794452      0.00000000
C      5.21678221      0.79120431      0.00000000
N      4.15308822      1.56345295      0.00000000
H     -0.42092938     -2.46773851      0.00000000
H      0.99421069     -3.52509463      0.00000000
H     -0.10742686     -0.18798815      0.00000000
H      5.52758959     -1.35729872      0.00000000
H      6.24625633      1.12769410      0.00000000
O     -2.28007418     -2.22739872      0.00000000
N     -1.68796951      2.34019804      0.00000000
N     -1.95907398      0.05644015      0.00000000
C     -3.94752687      1.46543329      0.00000000
C     -2.72640541     -1.06813199      0.00000000
N     -4.13680262     -0.88849832      0.00000000
C     -4.71981547      0.34599018      0.00000000
C     -2.51392107      1.28373237      0.00000000
H     -4.39339617      2.45391347      0.00000000
H     -4.69181635     -1.73813411      0.00000000
H     -5.80548489      0.37278718      0.00000000
H     -0.64857319      2.21087446      0.00000000
H     -2.07170720      3.27532304      0.00000000
```

G’C

E: −3436.78
H: −3314.89
G: −3349.49
N_{imag}: 1 (−69.0918)

```
N      0.61633338     -2.59086137      0.00000000
O      1.09171584      1.98444616      0.00000000
N      0.94408280     -0.30206626      0.00000000
C      1.55280904     -1.58866868      0.00000000
N      2.83888995     -1.67811771      0.00000000
C      1.63562954      0.86574410      0.00000000
H     -0.39606213     -2.42249286      0.00000000
H      0.93878657     -3.54764568      0.00000000
```

H	-0.09158596	-0.23464179	0.00000000
H	3.14304746	-2.65502699	0.00000000
H	2.72765172	0.73791405	0.00000000
C	-4.72958694	0.35297074	0.00000000
C	-2.52095751	1.28144559	0.00000000
H	-4.39547606	2.46014893	0.00000000
H	-4.70872850	-1.73049446	0.00000000
H	-5.81518163	0.38385192	0.00000000
H	-0.65841505	2.20633348	0.00000000
H	-2.07192476	3.27525656	0.00000000
O	-2.29634268	-2.22657578	0.00000000
N	-1.69189452	2.33890036	0.00000000
N	-1.96868093	0.05563897	0.00000000
C	-3.95311961	1.47009773	0.00000000
C	-2.73987379	-1.06817026	0.00000000
N	-4.15081576	-0.88266074	0.00000000

G’$_{isomer}$C

E: −3435.03
H: −3313.77
G: −3346.74
N_{imag}: 1 (−59.7858)

N	0.62894880	-2.56788401	0.00000000
O	1.07497478	2.01374586	0.00000000
N	0.96207724	-0.27962371	0.00000000
C	1.57261919	-1.58190406	0.00000000
N	2.83179888	-1.85442823	0.00000000
C	1.62344622	0.89899111	0.00000000
H	-0.38062111	-2.39903224	0.00000000
H	0.97377930	-3.51776930	0.00000000
H	-0.07646783	-0.21826139	0.00000000
H	3.42453873	-1.02544047	0.00000000
H	2.72368182	0.81617972	0.00000000
C	-4.73461593	0.34546843	0.00000000
C	-2.53517045	1.29432461	0.00000000
H	-4.41957104	2.45582543	0.00000000
H	-4.69218457	-1.73731430	0.00000000
H	-5.82046004	0.36590834	0.00000000
H	-0.68794960	2.24081971	0.00000000
H	-2.10935782	3.29497991	0.00000000
O	-2.27816047	-2.20991300	0.00000000
N	-1.71916523	2.36299627	0.00000000
N	-1.96796236	0.07561135	0.00000000
C	-3.96849553	1.46976059	0.00000000
C	-2.73053391	-1.05639066	0.00000000
N	-4.14339268	-0.88345151	0.00000000

GC’

E: −3723.94
H: −3601.27
G: −3637.91
N_{imag}: 0

N	0.60499468	-2.59927334	0.00000000
O	1.04135279	2.01302212	0.00000000
N	0.93355078	-0.29347753	0.00000000
C	1.47763874	-1.56053764	0.00000000
N	2.79075161	-1.78768224	0.00000000
C	3.51587113	-0.64769208	0.00000000
C	3.06780674	0.68939442	0.00000000
C	1.65665341	0.92269134	0.00000000
N	4.89292470	-0.55592632	0.00000000
C	5.21182018	0.80449437	0.00000000

N	4.14534208	1.57267330	0.00000000
H	−0.41183068	−2.47326576	0.00000000
H	0.99662412	−3.53046006	0.00000000
H	−0.10371072	−0.20156106	0.00000000
H	5.53221109	−1.34239061	0.00000000
H	6.23990059	1.14515513	0.00000000
O	−2.36918572	−2.22304034	0.00000000
N	−1.67533802	2.31538292	0.00000000
N	−1.91705983	0.02313129	0.00000000
C	−2.44001118	1.23223393	0.00000000
C	−2.76594423	−1.05782594	0.00000000
H	−2.10446653	3.23206780	0.00000000
H	−0.62144526	2.22367083	0.00000000
H	−3.52892017	1.39150861	0.00000000
H	−3.86010373	−0.83485699	0.00000000

G’C’

E: −2764.14
H: −2661.92
G: −2693.92
N_{imag} = 1 (−71.5630)

N	0.62843204	−2.57868305	0.00000000
O	1.07124222	1.99570166	0.00000000
N	0.95680208	−0.29140413	0.00000000
C	1.56803631	−1.57661387	0.00000000
N	2.85237339	−1.66508287	0.00000000
C	1.63236793	0.88303334	0.00000000
H	−0.38128018	−2.41062704	0.00000000
H	0.94592530	−3.53716310	0.00000000
H	−0.07962050	−0.23570683	0.00000000
H	3.16143833	−2.64032063	0.00000000
H	2.72619380	0.77452901	0.00000000
N	−1.92609253	0.02122705	0.00000000
C	−2.45159231	1.22619331	0.00000000
C	−2.78004876	−1.05973899	0.00000000
H	−2.12402148	3.23105709	0.00000000
H	−0.64503323	2.23029338	0.00000000
H	−3.54072023	1.38488799	0.00000000
H	−3.87347298	−0.83146402	0.00000000
O	−2.38601626	−2.22373869	0.00000000
N	−1.69188184	2.31611627	0.00000000

$G'_{isomer}C'$

E: −2762.26
H: −2660.07
G: −2692.33
N_{imag} = 1 (−60.6364)

N	0.63476257	−2.55961038	0.00000000
O	1.05327844	2.01986875	0.00000000
N	0.97216969	−0.27327157	0.00000000
C	1.58282097	−1.57477375	0.00000000
N	2.83973846	−1.84981989	0.00000000
C	1.61892510	0.91128218	0.00000000
H	−0.37199202	−2.39029737	0.00000000
H	0.97358413	−3.51172459	0.00000000
H	−0.06710347	−0.22247376	0.00000000
H	3.43522365	−1.02258139	0.00000000
H	2.71995856	0.84626342	0.00000000
N	−1.92302147	0.03482985	0.00000000
C	−2.46512073	1.23171973	0.00000000
C	−2.77262698	−1.05317928	0.00000000

H	-2.16901990	3.24325580	0.00000000
H	-0.67989014	2.26388388	0.00000000
H	-3.55623914	1.37439796	0.00000000
H	-3.86670756	-0.82810654	0.00000000
O	-2.37405811	-2.21397380	0.00000000
N	-1.72349905	2.33494527	0.00000000

AT

E: -4490.75
H: -4341.38
G: -4381.67
N_{imag} = 0

N	0.92035443	-0.38880954	0.00000000
C	1.53327667	-1.59393999	0.00000000
N	2.85048433	-1.84591750	0.00000000
C	3.56994435	-0.70836676	0.00000000
C	3.07250639	0.60637027	0.00000000
C	1.66288968	0.75165494	0.00000000
N	4.94772267	-0.55967961	0.00000000
C	5.20769043	0.80597294	0.00000000
N	4.10867230	1.53691264	0.00000000
N	1.03956757	1.94581976	0.00000000
H	0.86130749	-2.45119872	0.00000000
H	5.62272091	-1.31584513	0.00000000
H	6.21997993	1.19130904	0.00000000
H	1.60095564	2.78658973	0.00000000
H	0.01243177	2.00024760	0.00000000
N	-1.90237544	-0.36114457	0.00000000
C	-2.51620161	-1.60340854	0.00000000
N	-3.91727602	-1.52650728	0.00000000
C	-4.61809429	-0.33814473	0.00000000
C	-3.99742007	0.86960773	0.00000000
C	-2.53099287	0.88671810	0.00000000
O	-1.85881195	1.93357389	0.00000000
O	-1.91745208	-2.67468848	0.00000000
C	-4.73209331	2.18176831	0.00000000
H	-0.84260651	-0.37104968	0.00000000
H	-4.39908444	-2.41861172	0.00000000
H	-5.70061026	-0.43629644	0.00000000
H	-4.46157092	2.77934991	0.87913802
H	-5.81593829	2.02537457	0.00000000
H	-4.46157092	2.77934991	-0.87913802

A’T

E: -3525.90
H: -3397.35
G: -3432.97
N_{imag} = 1 (-38.8885)

N	0.93649743	-0.36049545	0.00000000
C	1.62273405	-1.58615412	0.00000000
N	2.90205962	-1.70353781	0.00000000
H	3.16646000	-2.69344491	0.00000000
H	2.72287215	0.74220226	0.00000000
C	1.62759850	0.74952384	0.00000000
N	1.03061259	1.94984496	0.00000000
H	0.92254765	-2.43109496	0.00000000
H	1.59226498	2.78953119	0.00000000
H	-0.00139330	2.02039069	0.00000000
H	-4.35787163	-2.43012756	0.00000000
H	-4.43548353	2.76697390	-0.87923691
H	-5.66576916	-0.45165522	0.00000000
H	-4.43548353	2.76697390	0.87923691

H	-5.78711801	2.00872279	0.00000000
N	-1.86533560	-0.36519657	0.00000000
C	-2.47712648	-1.60858284	0.00000000
N	-3.87920033	-1.53627016	0.00000000
C	-4.58347755	-0.35092193	0.00000000
C	-3.96535534	0.85836286	0.00000000
C	-2.49946786	0.87756931	0.00000000
O	-1.83333764	1.93099808	0.00000000
O	-1.87729481	-2.67916979	0.00000000
C	-4.70376190	2.16851089	0.00000000
H	-0.79778448	-0.37348316	0.00000000

A’$_{isomer}$T

E: -3522.06
H: -3393.64
G: -3429.58
N_{imag} = 1 (-133.8662)

N	0.93876606	-0.36285600	0.00000000
C	1.60330057	-1.61485906	0.00000000
N	2.84934692	-1.92125094	0.00000000
H	3.46613883	-1.09614252	0.00000000
H	2.70506952	0.79678782	0.00000000
C	1.60630245	0.75468743	0.00000000
N	0.99720994	1.95093442	0.00000000
H	0.87907984	-2.43446090	0.00000000
H	1.55017018	2.79653408	0.00000000
H	-0.03716615	2.01065624	0.00000000
H	-4.39011638	-2.41540954	0.00000000
H	-4.44396534	2.78208287	-0.87933879
H	-5.68802151	-0.43083736	0.00000000
H	-4.44396534	2.78208287	0.87933879
H	-5.79844200	2.02947323	0.00000000
N	-1.88813158	-0.36127941	0.00000000
C	-2.50538239	-1.60408417	0.00000000
N	-3.90706511	-1.52378942	0.00000000
C	-4.60527745	-0.33542395	0.00000000
C	-3.98177672	0.87139331	0.00000000
C	-2.51674742	0.88404704	0.00000000
O	-1.84571438	1.93500140	0.00000000
O	-1.90968778	-2.67528203	0.00000000
C	-4.71450322	2.18482347	0.00000000
H	-0.82595622	-0.37467351	0.00000000

AT’

E: -3449.34
H: -3337.68
G: -3373.22
N_{imag} = 0

N	0.92319688	-0.38883724	0.00000000
C	1.53816969	-1.59457480	0.00000000
N	2.85394769	-1.84475683	0.00000000
C	3.57351323	-0.70719571	0.00000000
C	3.07440961	0.60698957	0.00000000
C	1.66564155	0.75140683	0.00000000
N	4.95035488	-0.55694957	0.00000000
C	5.20846108	0.80900723	0.00000000
N	4.10857207	1.53870713	0.00000000
N	1.04692888	1.94938901	0.00000000
H	0.86846885	-2.45374767	0.00000000
H	5.62676554	-1.31206122	0.00000000
H	6.22019217	1.19571786	0.00000000
H	1.61165611	2.78810208	0.00000000

H	0.02449192	2.01437085	0.00000000
N	−1.88381559	−0.36615036	0.00000000
C	−2.57716872	−1.56994705	0.00000000
H	−3.68047000	−1.44192762	0.00000000
H	−0.81638897	−0.39131539	0.00000000
H	−3.61258623	0.80269789	0.00000000
C	−2.50573626	0.85731404	0.00000000
O	−1.91088297	1.93360964	0.00000000
O	−2.03578881	−2.66189441	0.00000000

A’T’

E: −2484.46
H: −2393.61
G: −2424.49
N_{imag} = 1 (−41.2369)

N	0.93350843	−0.38568056	0.00000000
C	1.61562726	−1.61623421	0.00000000
N	2.89299389	−1.73595451	0.00000000
H	3.15905245	−2.72527221	0.00000000
H	2.72699301	0.70154894	0.00000000
C	1.63221398	0.71928328	0.00000000
N	1.04972522	1.92723187	0.00000000
H	0.91314587	−2.45953506	0.00000000
H	1.62087646	2.76064023	0.00000000
H	0.02358615	2.01715564	0.00000000
H	−0.77585549	−0.40831267	0.00000000
O	−2.02795728	−2.67026870	0.00000000
H	−3.57468574	0.80036234	0.00000000
C	−2.46757852	0.84808705	0.00000000
O	−1.87060969	1.92559533	0.00000000
N	−1.85151494	−0.37554601	0.00000000
C	−2.55744074	−1.57269115	0.00000000
H	−3.65977058	−1.43380067	0.00000000

A’$_{isomer}$T’

E: −2480.36
H: −2389.63
G: −2420.81
N_{imag} = 1 (−134.2097)

N	0.94737060	−0.36040636	0.00000000
C	1.61309074	−1.61435540	0.00000000
N	2.85741799	−1.92083933	0.00000000
H	3.47774119	−1.09869760	0.00000000
H	2.71485442	0.79410611	0.00000000
C	1.61661128	0.75571845	0.00000000
N	1.01477447	1.95638278	0.00000000
H	0.88950315	−2.43479027	0.00000000
H	1.57187724	2.79938962	0.00000000
H	−0.01393796	2.02878908	0.00000000
H	−0.79344836	−0.39590003	0.00000000
O	−2.03640837	−2.65972853	0.00000000
H	−3.58648836	0.80889550	0.00000000
C	−2.47996519	0.85828080	0.00000000
O	−1.88468756	1.93708560	0.00000000
N	−1.86295386	−0.36434957	0.00000000
C	−2.56814716	−1.56487814	0.00000000
H	−3.67021197	−1.42560373	0.00000000

Guanine (G)

E: −2462.27
H: −2385.97
G: −2412.79
N_{imag} = 0

N	0.58390641	−2.61724469	0.27429696
O	1.02727007	2.01185338	−0.07649478
N	0.91954560	−0.29053357	0.12709885
C	1.46250444	−1.55061050	0.22380845
N	2.76162760	−1.78067783	0.21947344
C	3.50024460	−0.64558050	0.09271931
C	3.06667675	0.68698257	−0.00478067
C	1.65158201	0.96245033	0.00401962
N	4.87655127	−0.57668162	0.04738616
C	5.21093361	0.77200075	−0.07504138
N	4.15107345	1.55093701	−0.10888876
H	−0.31733405	−2.46677326	0.71425496
H	1.02990726	−3.49529487	0.51980778
H	−0.08998918	−0.17227896	0.08055378
H	5.50628815	−1.36976945	0.09360813
H	6.24177959	1.09869212	−0.13182183

N-acylguanidine (G')

E: −1505.80
H: −1449.48
G: −1473.07
N_{imag} = 0

N	0.53845114	−2.57397632	0.38579458
O	1.06716808	1.97169152	−0.15581006
N	0.92167094	−0.30425993	0.05985962
C	1.50912381	−1.57856577	0.21192525
N	2.78075378	−1.71563571	0.12474048
H	0.90774415	−3.49057758	0.61654084
H	−0.06943949	−0.24393008	−0.16002451
C	1.61435583	0.89969824	0.05200977
H	−0.27328422	−2.32369026	0.94480730
H	3.07592318	−2.67355737	0.33068852
H	2.68926956	0.76830880	0.25876371

Isomer of *N*-acylguanidine (G'_{isomer})

E: −1505.86
H: −1449.47
G: −1472.92
N_{imag} = 0

N	0.57380540	−2.53689463	0.43631141
O	1.03152134	2.00180976	−0.12959154
N	0.93639103	−0.29102193	−0.05965670
C	1.53027718	−1.56814564	0.14323777
N	2.77045225	−1.87206197	0.03631643
H	0.98735525	−3.44198020	0.64269543
H	−0.04379793	−0.25078851	−0.33395411
C	1.57013217	0.92871733	0.09053716
H	−0.16404919	−2.26705102	1.08056720
H	3.32943824	−1.11188416	−0.35609905
H	2.61279756	0.82997037	0.44963601

Cytosine (C)

E: −1904.41
H: −1839.97
G: −1864.40

N_{imag} = 0

H	-2.07137781	3.25724215	0.59007902
H	-0.71923301	2.16042339	0.56767359
O	-2.27319992	-2.21945668	-0.00177671
N	-1.69861407	2.34186398	0.38278615
N	-1.94563762	0.06599809	0.21318990
C	-3.94590582	1.47060240	0.14033356
C	-2.70189143	-1.07168618	0.05035853
N	-4.12729036	-0.86928125	-0.06172289
C	-4.71437125	0.35484799	-0.01619816
C	-2.52415987	1.25932023	0.25159600
H	-4.38876468	2.45991040	0.17179064
H	-4.67571785	-1.71572640	-0.17753738
H	-5.79640392	0.38847098	-0.11057224

(*E*)-*N*-(aminomethylene)formamide (C’)

E: −1232.42
H: −1187.61
G: −1209.42
N_{imag} = 0

O	-2.36215103	-2.20955515	-0.09582321
N	-1.71233724	2.33775424	0.36009651
N	-1.84763969	0.03544081	0.14662979
C	-2.41669848	1.19878849	0.19268791
C	-2.71797691	-1.04865141	-0.03064447
H	-0.70350417	2.28790536	0.45325179
H	-2.16782069	3.23882820	0.39362350
H	-3.50578293	1.34592565	0.10011756
H	-3.80420325	-0.77336753	-0.11215398

Adenine (A)

E: −2307.01
H: −2234.08
G: −2259.65
N_{imag} = 0

H	0.00874460	1.95059315	0.31125562
H	1.55871384	2.76636160	0.30458105
N	0.87949712	-0.40391670	0.08900425
C	1.50184706	-1.59781857	0.00804349
N	2.82254608	-1.85191109	-0.04730707
C	3.54240364	-0.71910242	-0.01158843
C	3.03998559	0.59271536	0.07031421
C	1.63264955	0.71817954	0.11886488
N	4.91998363	-0.56300738	-0.04153450
C	5.17379832	0.80192010	0.02389694
N	4.07349496	1.52768990	0.09201141
N	1.01106860	1.92632125	0.17813530
H	0.84642963	-2.46771636	-0.01385566
H	5.59890907	-1.31335090	-0.09901656
H	6.18443109	1.19166130	0.01719508

(*E*)-*N*-(aminomethylene)formimidamide (A’)

E: −1341.61
H: −1288.88
G: −1311.04
N_{imag} = 0

H	3.16602928	-2.74010342	0.11991935

N	0.92818114	−0.42994519	0.13473718
C	1.63222793	−1.62317577	−0.02549478
N	2.86282313	−1.77968218	0.31706894
H	2.63659437	0.71660459	−0.28782161
C	1.58940529	0.67614370	0.03903789
N	1.03295739	1.87810459	0.33288203
H	1.00353299	−2.42847670	−0.43140163
H	1.52793651	2.73920817	0.15292519
H	0.07048875	1.91903963	0.64814744

Isomer of (*E*)-*N*-(aminomethylene)formimidamide (A'_{isomer})

E: −1339.79
H: −1287.10
G: −1309.14
N_{imag}: 0

N	0.95940404	−0.37082676	−0.03564023
C	1.61551618	−1.60517982	0.06160983
N	2.79204682	−1.93306975	−0.33458695
H	3.27071422	−1.12783073	−0.76572313
H	2.53809939	0.70198833	0.84123100
C	1.56423903	0.70396612	0.32855291
N	1.05504966	1.94924163	0.10446986
H	0.98132117	−2.39175593	0.48578413
H	1.43176393	2.74639007	0.59845174
H	0.12140806	2.02684032	−0.28414916

Thymine (T)

E: −2167.23
H: −2092.10
G: −2118.23
N_{imag} = 0

N	−1.86964944	−0.33090681	0.00000000
C	−2.46167609	−1.59088017	0.00000000
N	−3.85864620	−1.52017012	0.00000000
C	−4.57287282	−0.33529469	0.00000000
C	−3.97399287	0.88186150	0.00000000
C	−2.50511969	0.93759711	0.00000000
H	−4.33459840	−2.41525299	0.00000000
H	−4.46830426	2.78284601	−0.87905649
H	−5.65362959	−0.45008702	0.00000000
O	−1.83244310	1.96566984	0.00000000
O	−1.84333134	−2.64755127	0.00000000
H	−4.46830426	2.78284601	0.87905649
H	−5.81137935	2.00764977	0.00000000
C	−4.73041353	2.18156958	0.00000000
H	−0.85173624	−0.32150487	0.00000000

N-formylformamide (T')

E: −1126.05
H: −1088.61
G: −1109.93
N_{imag} = 0

N	−1.82921464	−0.32454075	0.00000000
C	−2.47269296	−1.56141386	0.00000000
H	−3.57712878	−1.46525247	0.00000000
O	−1.89286458	1.97780211	0.00000000
H	−3.57967093	0.81226734	0.00000000
C	−2.47545307	0.91089314	0.00000000
H	−0.80782094	−0.32340102	0.00000000
O	−1.88772430	−2.62701897	0.00000000
